# Model-informed development of bacteriophage therapy: bridging *in vitro* and *in vivo* efficacy against multidrug-resistant *Pseudomonas aeruginosa*

**DOI:** 10.1128/msystems.01384-25

**Published:** 2025-11-13

**Authors:** Jun Seok Cha, Kyungnam Kim, Hwa Jeong You, Dasom Kim, Hyun Hee Park, SuJin Heo, Choon Ok Kim, Byung Hak Jin, Dongeun Yong, Dongwoo Chae

**Affiliations:** 1 Department of Pharmacology, Yonsei University 26721, Seodaemun-gu, Seoul, Republic of Korea; 2 Department of Pharmacology, Graduate School of Medical Science, Brain Korea 21 Project, Yonsei University College of Medicine 37991, Seodaemun-gu, Seoul, Republic of Korea; 3 Microbiotix Co., Ltd, Seoul, Republic of Korea; 4 Department of Clinical Pharmacology, Severance Hospital, Yonsei University Health System, Seoul, Republic of Korea; 5 Department of Laboratory Medicine and Research Institute of Bacterial Resistance, Yonsei University College of Medicine, Seoul, Republic of Korea; Rice University, Houston, Texas, USA

**Keywords:** bacteriophage therapy, predator-prey dynamics, pharmacokinetics pharmacodynamics, mathematical modeling, *Pseudomonas aeruginosa*, bacteriophages, computational biology, phage cocktail optimization, model-informed drug development, quantitative systems pharmacology

## Abstract

Bacteriophages are emerging as promising alternatives to antibiotics for multidrug-resistant (MDR) infections. However, their unique pharmacokinetic and pharmacodynamic (PKPD) properties arising from host-dependent amplification present challenges for dose selection and clinical translation. Here, we present a mechanistic PKPD model informed by *in vitro* kinetic assays and *in vivo* mouse studies of phage therapy targeting MDR *Pseudomonas aeruginosa*. The model extends the classical predator-prey model by addressing dormancy-related bacterial persistence and partitioning bacterial subpopulations based on phage susceptibility profiles. Simulations revealed a non-monotonous dose-exposure curve driven by dose-dependent reduction of phage replication and the importance of cross-resistance in selecting optimal phage cocktails. *In vivo*, host immunity was identified as a crucial component in inhibiting bacterial regrowth, with bistable outcomes dependent on initial bacterial load and immune competence. Dose-ranging simulations under varying immune statuses suggest that long-term bacterial load is solely determined by host immune function. However, higher doses transiently reduce bacterial load to a greater extent and thereby suppress immune activation. In immunocompetent hosts, phage cocktails can enhance maximal bacterial load reduction when administered at doses higher than a critical threshold. In conclusion, our PKPD framework enables optimal selection of phage cocktails and dosing regimens, supports rational design of first-in-human trials of phage therapy, and potentially advances model-informed drug development for replication-competent biologics.

**IMPORTANCE:**

In this study, we construct an integrative model of phage-bacteria dynamics and investigate whether its calibration to *in vitro* kinetic assay data can inform the rational design of phage therapy regimens and cocktails. Our findings demonstrate a dose range within which lower phage doses yield higher long-term exposure, presenting a fundamentally different framework for dose optimization. Analysis of phage cocktails reveals that combining phages with low cross-resistance delays the regrowth of phage-resistant bacteria *in vitro*. The extended *in vivo* model elucidates key differences between *in vitro* and *in vivo* outcomes and highlights the importance of the host’s immune response in suppressing the growth of phage-resistant bacteria. Phage cocktails to combat phage resistance are therefore of less importance in immune-competent individuals but can enhance bacterial killing when administered at sufficiently high doses. We propose that this modeling framework holds potential for model-informed drug development by quantitatively characterizing bacteria-phage dynamics using preclinical data. Furthermore, it may facilitate the interpretation of *in vivo* therapeutic outcomes through a mechanistic understanding derived from *in vitro* observations.

## INTRODUCTION

Multidrug-resistant (MDR) *Pseudomonas aeruginosa* is classified as a “priority 1: critical” pathogen by the World Health Organization due to its increasing resistance to available treatments (1). In response, clinicians have revisited older antibiotics, optimized dosing strategies, and introduced novel antimicrobial agents (2–7). However, resistance to these newer therapies is already emerging, underscoring the urgent need for alternative approaches (8, 9).

Bacteriophage (phage) therapy has re-emerged as a promising strategy, supported by anecdotal clinical successes and evidence from animal models (10–13). Notably, prior studies have demonstrated the efficacy of phage therapy in murine models of bacterial infections including *P. aeruginosa* pneumonia (12, 14–16), and several recent investigations have characterized pharmacokinetics (PK) and therapeutic outcomes of phage cocktails administered intravenously or by inhalation (17).

Yet, translating phage therapy into clinical practice is challenging due to its unique pharmacology, characterized by self-amplification, bacterial host dependence, and complex interactions with the host immune system (14). Higher phage doses, or multiplicities of infection (MOIs), are reportedly associated with accelerated bacterial resistance (18). Phenotypic persistence arising from bacterial dormancy is another aspect to consider when optimizing treatment doses and timing (19).

Important challenges in phage cocktail design include phage-phage competition, wherein a more virulent phage suppresses the replication of a companion phage (20), and cross-resistance between phages targeting different bacterial receptors, complicating predictions of the combined effect of multiple phages (21). Despite these insights, the field lacks a quantitative framework that integrates these factors—bacterial population heterogeneity, phage-bacteria interaction kinetics, cross-resistance, phage-phage interactions, and host immune responses—to inform rational cocktail composition, dosing strategies, and timing of administration.

In this study, we sought to address this gap by developing an integrated experimental and mathematical modeling framework that characterizes *in vitro* and *in vivo* dynamics of phage therapy for MDR *P. aeruginosa*. Our approach extends classical predator-prey models to incorporate dormant bacterial states, strain-specific phage susceptibility, and immune clearance, with the goal of supporting rational design of phage cocktails and optimized treatment regimens for future clinical trials.

## MATERIALS AND METHODS

### Experimental model details

#### Phages and bacterial strains

Three phages—MP-A, PP-A, and PP-B—were isolated for therapeutic use from hospital sewage (Severance Hospital, Seoul, Korea). Transmission electron microscopy (TEM) analysis classified MP-A as *Myoviridae* and PP-A/PP-B as *Podoviridae* (Fig. S1A). Receptor profiling showed that MP-A and PP-B target lipopolysaccharides, while PP-A targets pili. Lytic spectra against domestic *P. aeruginosa* YMC strains were 60% (MP-A), 56% (PP-A), and 54% (PP-B) (Fig. S1B). Genomes ranged from 40–70 kb with 50-100 predicted genes (Fig. S1C). Adsorption and one-step growth assays revealed strain-specific differences in infection kinetics (Fig. S1D and E).

The carbapenem-resistant *P. aeruginosa* 15-4 strain, resistant to both imipenem and meropenem, was isolated from a clinical pneumonia case. An additional 12 strains with various antibiotic resistance profiles were collected similarly. Detailed collection protocols are described in the Supplementary Methods.

#### Animal model

Male and female ICR mice (5–7 weeks old) were used for *in vivo* experiments. The type of mice used for each experiment is detailed for each experiment in the Supplementary Materials. Mice were housed under controlled conditions with *ad libitum* access to food and water. All animal experiments were conducted in accordance with institutional guidelines and were approved by the Institutional Animal Care and Use Committees (IACUCs) of the respective facilities: HDS Bio, Pohang-si, Republic of Korea (IACUC ID: 20231024-21); DT&CRO, Yongin-si, Republic of Korea (IACUC ID: 23E019); and HLB Biostep, Incheon, Republic of Korea (IACUC ID: 24-HB-0126). Mice were intranasally inoculated with 20 μL of *P. aeruginosa* 15-4, followed one or 2 h later by intravenous phage administration via the tail vein. Full experimental details are available in the Supplementary Methods.

### Method details

#### Bacteriophage characterization and *in vitro* assays

Phage genomes were sequenced, assembled (SPAdes v3.15.0) (22), and annotated (Prokka v1.14.6) (23). Quality control (Trimmomatic v0.36, QUAST v5.0.2) (24, 25), tRNA prediction (tRNAscan-SE v2.0.7) (26), and TEM-based morphological classification followed ICTV guidelines. Adsorption and one-step growth assays quantified infection kinetics.

*In vitro* kinetic assays were conducted to explore bacteria-phage dynamics. Host bacteria were incubated and inoculated into Luria-Bertani medium in 96-well plates (with a total volume of 100 µL per well) or 25 mL flasks, with $OD_{600}$ measurements taken hourly for 24 h. Bacteria were added to each well to achieve a starting concentration of 5.0 ×$10^{6}$ CFU/mL. Phage treatments (both single and cocktail) were added at 50 µL per well to achieve designated MOI values (ranging from 10^−7^ to 10^2^).

To evaluate dormancy, cultures were grown in Mueller-Hinton broth and assayed using $OD_{590}$ and a redox-based metabolic activity assay. To assess phage infectivity in dormant populations, cultures were pre-incubated in M9 medium for 48 h prior to MP-A or PP-A addition. CFU and PFU were measured every 24 h for 7 days post-infection.

Detailed protocols for bacteriophage characterization and *in vitro* assays are provided in the Supplementary Methods.

#### *In vivo* mouse PKPD experiment and PK analysis

As a baseline, mice were intranasally inoculated with *P. aeruginosa* at $10^{6}$, $10^{7}$, or $5\times 10^{7}$CFU/mouse (*n* = 10/group). Survival was monitored every 6 h to assess dose-dependent lethality.

The PK of the MP-A + PP-A cocktail were evaluated in uninfected mice, while both PK and pharmacodynamics (PD) were assessed in infected mice. Mice received $10^{7}$, $10^{9}$, or $10^{11}$ PFU/mouse, with three to four mice per time point per group. The interval from bacterial infection to phage treatment was 1 h.

Detailed protocols for *in vivo* PKPD analysis are provided in the Supplementary Methods.

#### *In vitro* bacteria-phage population dynamics modeling and parameter estimation

The dynamics of the *in vitro* phage-bacteria interactions were described by a system of ordinary differential equations (ODEs) assuming mass-action kinetics in a well-mixed environment. The model incorporated wild-type bacteria (W) and phage-refractory mutants (M), with each bacterial subpopulation switching between a proliferative, phage-sensitive state (*P*), and a dormant, phage-refractory state (D).

We first developed a simplified single-phage model (hereafter referred to as the *in vitro* single-phage model) to characterize the basic population dynamics and identify influential parameters. A local sensitivity analysis was performed by perturbing each parameter by ±20% and examining the resulting changes in key output metrics: maximum bacterial suppression, peak phage amplification, and total bacterial burden at 24 h.

We extended the *in vitro* single phage model to a model incorporating all three phages (*in vitro* phage cocktail model). All possible resistance combinations to MP-A, PP-A, and PP-B were considered. Parameters for the *in vitro* phage cocktail model were estimated using Monolix v2023R1 via non-linear mixed-effects modeling.

Data for parameter estimation were obtained from 96-well *in vitro* kinetic assays across a range of phage doses. Independent experiments conducted in 25 mL flasks were used for model validation.

Additional model details are in the “Mathematical modeling of *in vitro* phage-bacteria interactions” section in the Results and Supplementary Methods.

#### Application of the *in vitro* phage cocktail model to diverse clinical isolates

To evaluate generalizability, the model was applied to 12 additional clinical *P. aeruginosa* strains exposed to MP-A, PP-A, and their combination at MOIs of 0.01, 1, and 100. Each assay began with $5.0\times 10^{6}$CFU/mL. Model parameters were estimated for each strain using Monolix v2023R1 (Lixoft, Antony, France). Hierarchical clustering based on fitted parameters was performed to classify strains by virulence profiles. Modeling details are provided in the Supplementary Methods.

#### Extension of the *in vitro* phage cocktail model to *in vivo*

The original *in vitro* model was extended to incorporate phage PK and host immunity and recalibrated using PKPD data from infected mice. Details are available in the “Model extension for *in vivo* conditions” section in the Results and the Supplementary Methods.

#### *In vivo* mouse lung bacterial load experiment

Two experiments assessed relative efficacy of phage cocktails and dose response on lung bacterial burden:

i) Relative efficacy of phage cocktail: Control (*n* = 6), MP-A, PP-A, or MP-A + PP-A (all at $5\times 10^{9}$ PFU/mouse, *n* = 6 each).

ii) Dose response: Control (*n* = 5), MP-A + PP-A at $10^{7}$ or $10^{11}$ PFU/mouse (*n* = 5/group).

The interval from bacterial infection to phage treatment was 2 h. Detailed methodologies are provided in the Supplementary Methods.

#### Evaluating the efficacy of the phage cocktail

An acute pneumonia mouse model was established by intranasally inoculating mice with $5\times 10^{8}$CFU/mouse of *P. aeruginosa*.

Control group: Received 0.2 mL saline via tail vein injection.

Treatment groups: Received MP-A + PP-A cocktail at $5\times 10^{4}$, $5\times 10^{6}$, $5\times 10^{8}$, and $5\times 10^{10}$ PFU/mouse via tail vein injection.

Each group contained eight replicates, and survival was assessed at 0, 24, 48, 72, and 96 h. The interval from bacterial infection to phage treatment was 2 h. Detailed methodologies are provided in the Supplementary Methods.

#### Quantification and statistical analysis

Details of quantification and statistical analysis are provided in the figure legends. Analysis of variance (ANOVA) and Tukey’s honestly significant difference (HSD) test were performed using SciPy v1.11.2 (27). Survival analysis was conducted using the log-rank test, and Kaplan-Meier survival probability estimates were computed using Lifelines v0.27.7 (28). Non-linear mixed-effects modeling of *in vitro* PKPD data were conducted using Monolix v2023R1 (Lixoft, Antony, France).

## RESULTS

### *In vitro* dynamics of mono- and cocktail phage treatments

**Fig 1:**
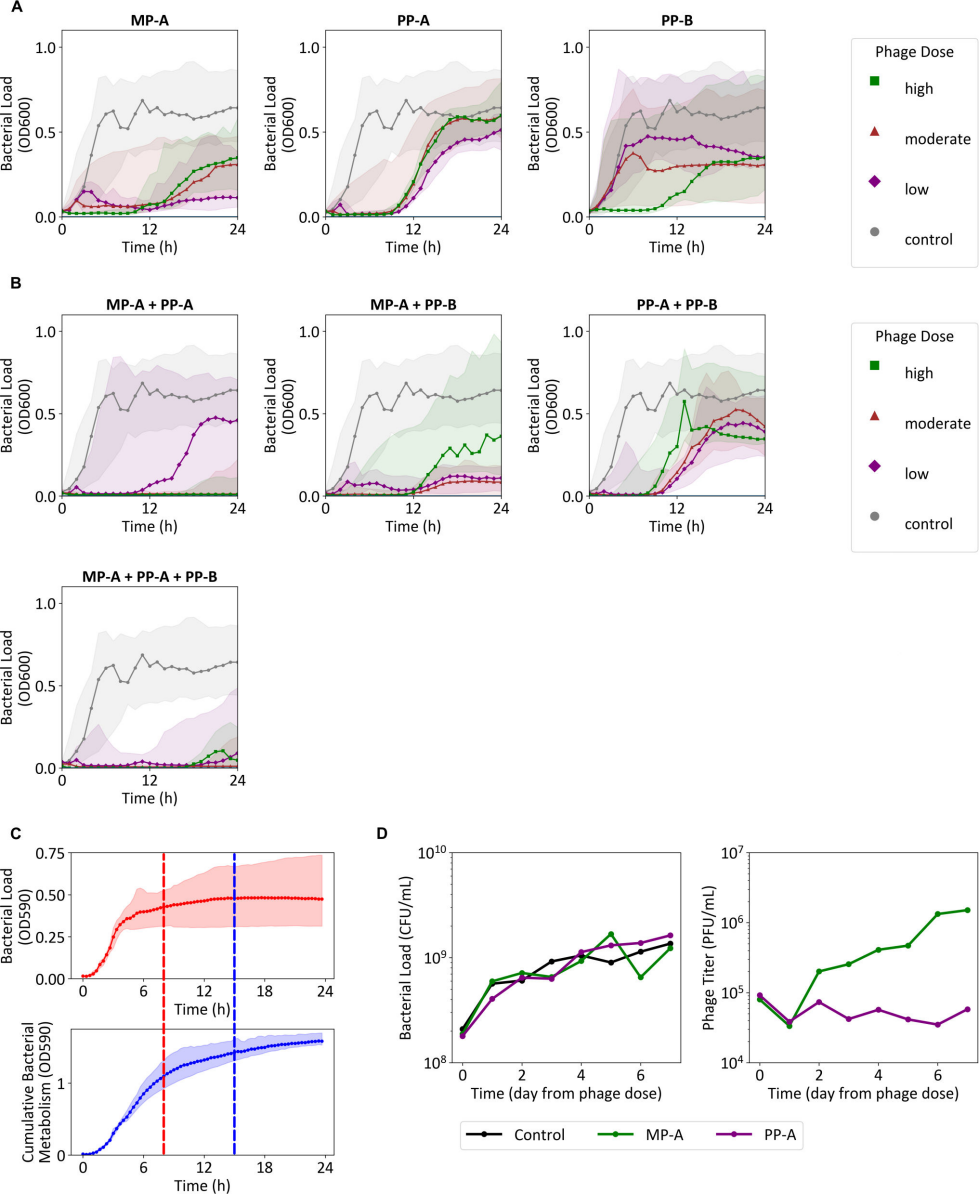
*In vitro* kinetic assays and dormant bacteria characterization. *In vitro* kinetic assays for (**A**) single phage or (**B**) phage cocktail were conducted for all treatments, with results grouped into dose cohorts: control (no dose), low dose (MOI 10^−7^−10^−5^), moderate dose (10^−4^−10^−1^), and high dose (10^0^−10^2^). The replicate numbers for each MOI are presented in Table S4. The median is shown as a solid line, surrounded by a band from the 5th to the 95th percentile. The same control group is plotted for each subplot. (**C**) Bacterial growth (red, *n* = 9) and cumulative bacterial metabolism (blue, *n* = 9) from the dormant bacteria formation assay are presented. The median is shown as a solid line, surrounded by a band from the 5th to 95th percentile. The time points when the bacterial growth and cumulative bacterial metabolism reach steady state, defined here as 90% of the values of measurements at 24 h are shown as vertical dashed lines. (**D**) Bacterial load and phage titers for MP-A and PP-A are shown for the dormant bacteria kinetic assay.

In 24-h *in vitro* kinetic assays against MDR strain 15-4 using MP-A, PP-A, PP-B, and all possible phage combinations, repeated over a wide range of phage doses spanning MOI of $10^{-7}$ to $10^{2}$, all phages achieved complete suppression at MOI 1. However, regrowth was observed after 10 h across all conditions (Fig. 1A). PP-A showed the strongest initial potency, rapidly achieving complete bacterial load suppression even at low MOIs, followed by MP-A and PP-B. Across all phages, higher MOIs paradoxically accelerated regrowth, suggesting stronger selection pressure favoring phage-resistant mutants.

While MP-A + PP-B and PP-A + PP-B failed to suppress the emergence of resistant bacteria, MP-A + PP-A significantly prolonged bacterial suppression compared to individual phages (Fig. 1B). The three-phage cocktail offered no clear benefit over MP-A + PP-A in delaying bacterial regrowth given moderate to high phage doses. At low doses, the three-phage cocktail suggested superior regrowth inhibition of phage-resistant bacteria (Welch’s *t*-test; *P* = 0.03); however, there was no statistically significant difference of 24-h bacterial load between the moderate (Welch’s *t*-test; *P* = 0.78) and highest (Welch’s *t*-test; *P* = 0.14) dose cohort of the two cocktails.

### Dormant bacteria formation and its tolerance to phage infection

We next sought to understand why low phage doses of MP-A and PP-B failed to induce complete bacterial suppression prior to the regrowth phase. We hypothesized that this was due to the delayed onset of bacterial decline at lower doses that subsequently resulted in a higher fraction of phage-tolerant dormant bacteria. Simultaneous monitoring of cellular respiration (via NADH production) and growth (via turbidity) using the Odin platform (Biolog, Inc., Hayward, CA, USA) indeed showed a gradual decline in NADH-linked redox activity as cultures approached stationary phase, indicating dormancy (Fig. 1C).

To verify whether dormant bacteria are unaffected by phages, we applied MP-A and PP-A to deep-dormant populations (29) induced by prolonged (>48 h) starvation. The results revealed that neither phage exhibited propagation or killing activity, confirming dormancy-mediated tolerance (Fig. 1D).

Collectively, these results suggest that low phage doses, due to their slower bacterial killing, allow greater formation of dormant bacteria. As a result, phages fail to eradicate bacteria that are genetically susceptible but phenotypically tolerant.

### *In vivo* PK/PD evaluation

#### The impact of bacterial inoculum size on host survival

**Fig 2:**
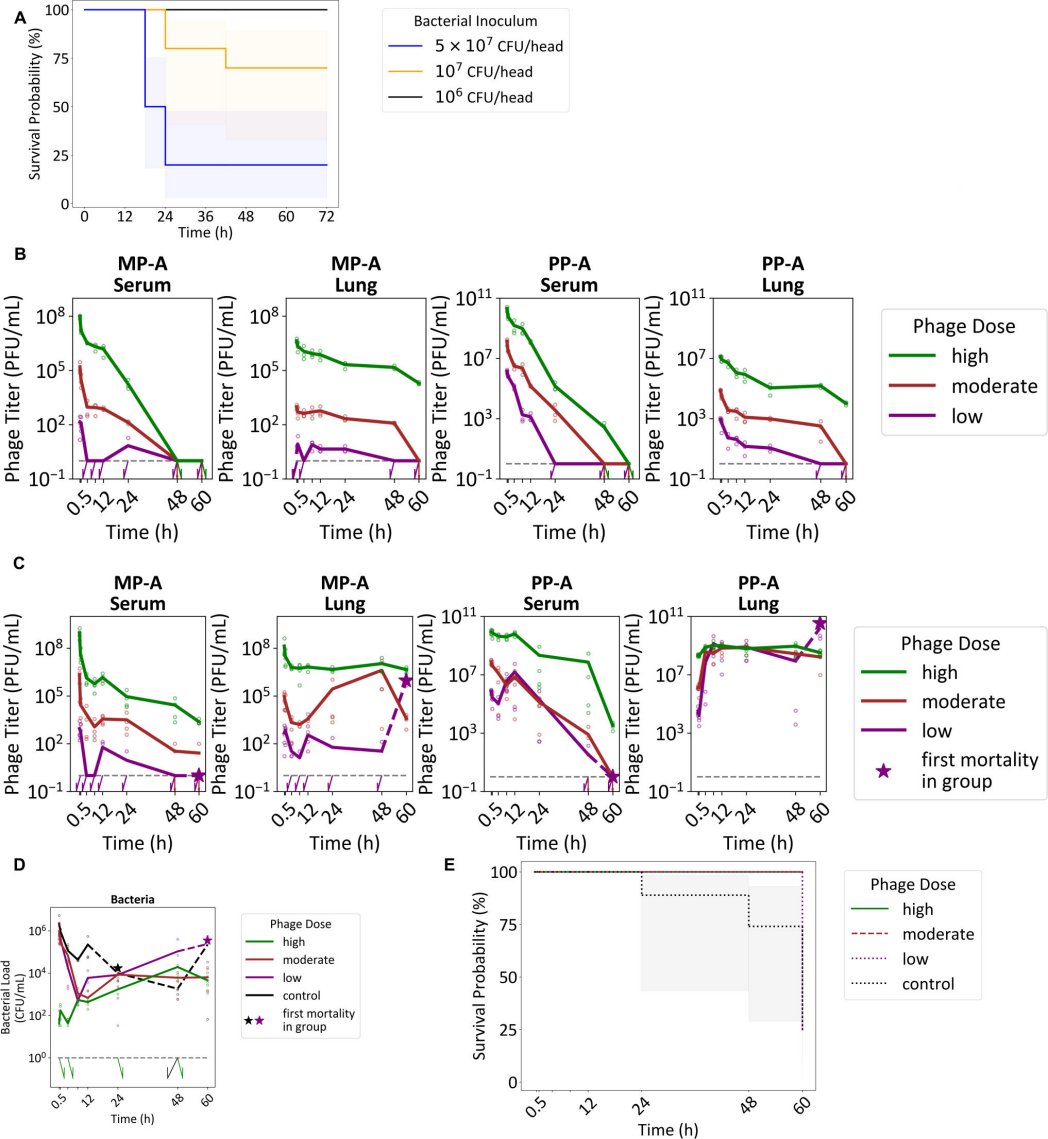
*In vivo* phage PKPD and efficacy. (**A**) The Kaplan-Meier plot depicts the survival of mice in relation to varying levels of bacterial load. PK profiles in the (**B**) non-infected model and PKPD profiles in the (**C–D**) acute pneumonia model are shown. The dosing groups used are low (10^7^ PFU/head), moderate (10^9^ PFU/head), and high (10^11^ PFU/head). Measurements below the Lower Limit of Quantification (LLOQ) (phage: 1 PFU/mL, bacteria: 1 CFU/mL, gray dashed line) were imputed with the LLOQ value when calculating the mean for each respective time point. The means for each time point are connected with solid lines for time points before the first mortality in dosing group and with dashed lines for the time points after the first mortality in dosing group. (**E**) The Kaplan-Meier plot illustrates the survival of mice based on the dosage of administered phage.

Mice inoculated intranasally with *P. aeruginosa* 15-4 showed dose-dependent mortality. All survived at $\leq 10^{6}$CFU/mouse; survival dropped to about 80% and 30% at $10^{7}$ and $5\times 10^{7}$CFU/mouse, respectively (Fig. 2A).

### PK of MP-A + PP-A in uninfected mice

We evaluated phage PK at doses of $10^{7}$, $10^{9}$, and $10^{11}$ PFU/mouse (Fig. 2B and C). MP-A exhibited non-linear kinetics, with dose-normalized $C_{0}$ and $AUC_{last}$ increasing with dose (Table S4). PP-A displayed linear PK, achieving 2–5 log higher serum concentrations than MP-A at equivalent doses. However, MP-A showed superior lung penetration, with a higher lung/serum $C_{0}$ ratio.

### PKPD analysis in infected mice

Using a $10^{7}$CFU/mouse challenge, phages were administered i.v. 2-h post-infection. MP-A again showed dose-dependent increases in serum $C_{0}/dose$ and $AUC_{last}/dose$. In the lungs, $AUC_{last}/dose$ decreased at higher doses, indicating suppressed replication. This trend was supported by a declining infected/uninfected AUC ratio with increasing dose (Table S5).

For PP-A, serum PK remained dose-independent, but lung $AUC_{last}/dose$ decreased with increasing dose. The infected/uninfected lung AUC ratio for PP-A was 10×30 × higher than for MP-A, indicating greater *in vivo* amplification.

Phage treatment produced an early bacterial decline followed by a modest rebound. Unlike *in vitro* assays, bacterial loads stabilized near $10^{4}$CFU/mL by 24 h (Fig. 2D). Survival was significantly improved at $10^{9}$ and $10^{11}$ PFU/mouse (*P* = 0.006), but not at $10^{7}$ PFU/mouse (*P* = 0.3) (Fig. 2E).

### Mathematical modeling

#### Overview of the model construction process

**Fig 3:**
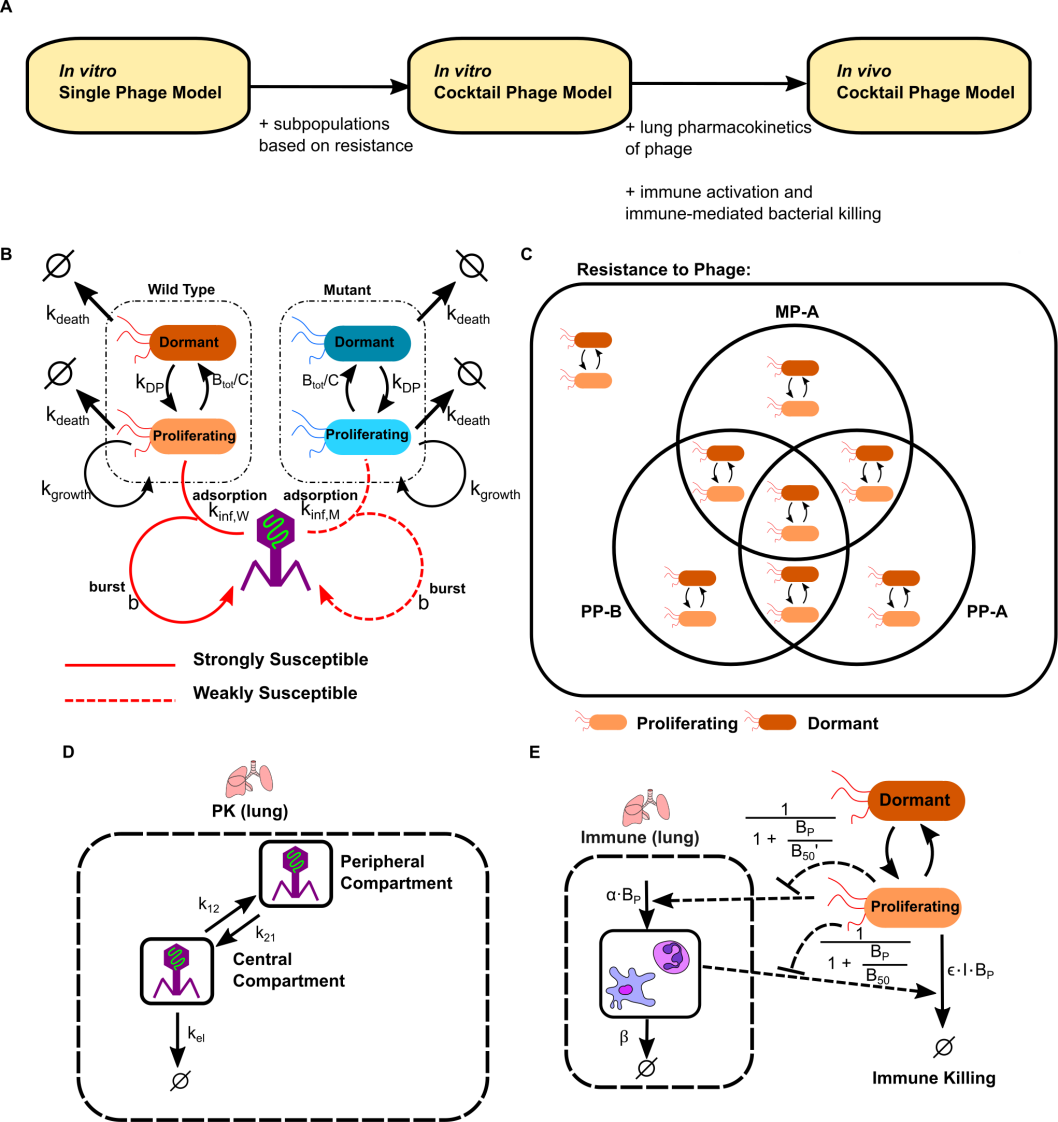
Overview of the modeling framework. (**A**) Stepwise development proceeding from a single-phage *in vitro* model to a multi-phage cocktail model and finally to the *in vivo* model incorporating PK and host immunity. (**B**) Schematic representation of the *in vitro* single-phage model. (**C**) Subpopulation structure illustrating all possible combinations of resistance to MP-A, PP-A, and PP-B, used in the cocktail model. (**D**) Model diagram of phage PK in the lung, represented by a two-compartment structure. (**E**) Schematic of the innate immune module, showing bacterial burden-dependent immune activation and immune-mediated clearance of proliferating bacteria.

The development of the phage-bacteria-host dynamic model followed a stepwise approach (Fig. 3A). We began by constructing an *in vitro* single-phage model, which categorized bacterial subpopulations according to their resistance status to a single phage (Fig. 3B). This model was used to simulate time-course bacterial dynamics and to perform sensitivity analysis, identifying key parameters that significantly influenced bacterial suppression and phage amplification.

Next, the model was extended into an *in vitro* phage cocktail model, incorporating all three phages (MP-A, PP-A, and PP-B) and all possible resistance combinations among bacterial subpopulations (Fig. 3C). Model parameters were estimated using data from *in vitro* kinetic assays across multiple MOI conditions, as described in the Materials and Methods.

Finally, we expanded the *in vitro* cocktail model to build an *in vivo* phage cocktail model by incorporating two additional components: phage PK (Fig. 3D) and innate immune-mediated bacterial clearance (Fig. 3E). These new modules were parameterized and calibrated using *in vivo* PK/PD data from mouse infection models, enabling simulation of dose-dependent bacterial and phage dynamics under varying immune conditions.

### *In vitro* single-phage model development

To integrate experimental findings, we developed an ODE-based model incorporating wild-type (W) and mutant (M) bacterial variants, each capable of switching between proliferating (*P*) and dormant (D) states. The following equations describe the dynamics of wild-type, proliferating bacteria ($B_{W,P}$), wild-type, dormant bacteria ($B_{W,D}$), mutant, proliferating bacteria ($B_{M,P}$), and mutant, dormant bacteria ($B_{M,D}$) against a single phage (*P*) (Fig. 3B).


(1) $$\begin{array}{c} \frac{d}{dt}B_{W,P}=k_{growth}\cdot B_{W,P}-k_{inf,W}\cdot B_{W,P}\cdot P\;-k_{PD}\cdot B_{W,P}+k_{DP}\cdot B_{W,D}\;-k_{death}\cdot B_{W,P} \end{array}$$

(2) $$\begin{array}{c} \frac{d}{dt}B_{W,D}=k_{PD}\cdot B_{W,P}-\;k_{DP}\cdot B_{W,D}-k_{death}\cdot B_{W,D} \end{array}$$

(3) $$\begin{array}{c} \frac{d}{dt}B_{M,P}=k_{growth}\cdot B_{M,P}-k_{inf,M}\cdot B_{M,P}\cdot P\;-k_{PD}\cdot B_{M,D}+k_{DP}\cdot B_{M,D}\;-k_{death}\cdot B_{M,P} \end{array}$$

(4) $$\begin{array}{c} \frac{d}{dt}B_{M,D}=k_{PD}\cdot B_{M,P}-\;k_{DP}\cdot B_{M,D}-k_{death}\cdot B_{M,D} \end{array}$$

Key model parameters included bacterial growth rate ($k_{growth}$), distinct phage infectivity constants for wild-type ($k_{inf,W}$) and mutant ($k_{inf,M}$) strains, and transition rates between proliferative and dormant states ($k_{PD}$ and $k_{DP}$). Dormancy was modeled as a density-dependent process, and cocktail treatments were implemented by extending the model to track multiple phages and bacterial subpopulations with various resistance profiles (Fig. 3C). The full set of equations and parameter definitions is provided in the Supplementary Methods.

### Local sensitivity analysis

To assess parameter influence, we performed local sensitivity analysis by perturbing each parameter by ±20% and quantifying its effect on three outcome metrics: minimum bacterial load, 24-h bacterial load, and 24-h phage load. Baseline parameter values were selected to produce a canonical infection time course characterized by early bacterial growth, phage-mediated suppression of sensitive populations, and regrowth of resistant bacteria (Fig. S2A).

Key findings from the sensitivity analysis (Fig. S2B through D) included:

- Higher wild-type infection rate constant decreased minimum bacterial load but paradoxically increased 24-h bacterial load, suggesting faster selection of phage-resistant mutants.
- Higher mutant infection rate constant had negligible effects on minimum bacterial load but decreased 24-h bacterial load and increased 24-h phage load. This shows that mutant infection rate majorly affects the later phase of the kinetic curve, where mutant bacterial strains are dominant.
- Higher proliferative carrying capacity increased the 24-h bacterial load and 24-h phage load but decreased the minimum bacterial load. The decrease in minimum bacterial load is due to slower entry into dormancy when the proliferative capacity is higher.

### *In vitro* cocktail model development

**Fig 4:**
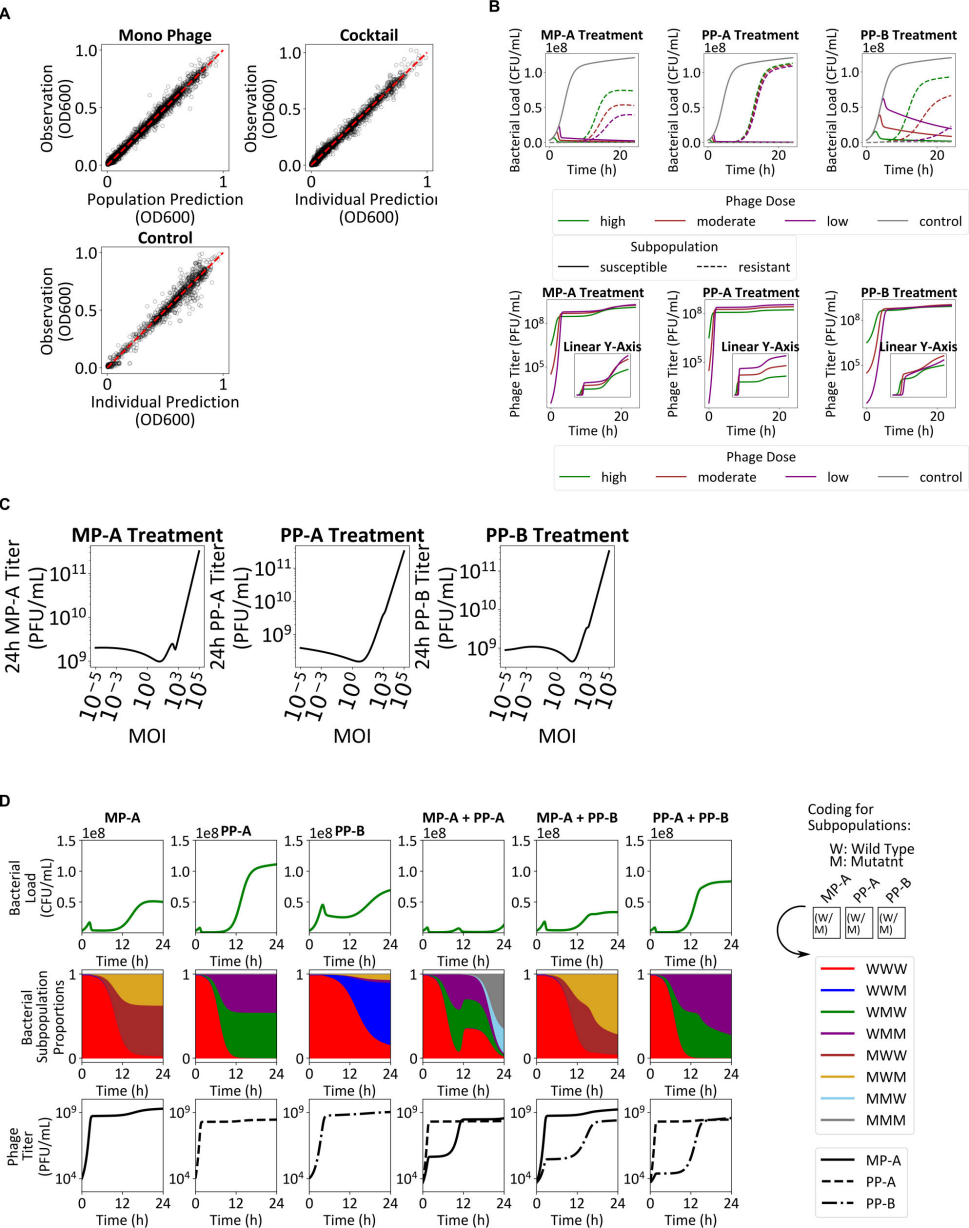
Simulations of bacteria-phage dynamics. (**A**) Observation vs individual prediction goodness-of-fit plots are presented, for the mono phage, cocktail, and control groups. (**B**) A dose-response simulation of the MP-A + PP-A cocktail in control and MOI of 0.0001, 0.01, and 1. For the bacteria titer, bacteria susceptible to the treatment (solid line) and resistant to the treatment (dashed line) are plotted separately. For the phage titer, the inset shows the plot in linear *y*-scale. (**C**) Based on dose-response simulations for MP-A, PP-A, and PP-B treatments, the 24-h phage titer vs MOI are shown. (**D**) The temporal dynamics of bacterial subpopulation fractions were analyzed to evaluate single-phage treatments and double-phage cocktails. Rows 1–3 illustrate the time-course profiles of the total bacterial load, proportions of bacterial subpopulations, and phage titers, respectively.

We extended the single phage model to a model incorporating multiple phages, whose details are presented in the Supplementary Methods. The model was calibrated to *in vitro* kinetics, accurately capturing bacterial suppression and regrowth patterns (Fig. 4A). Parameter estimates are summarized in Table 1, with inter-experimental variances in Table S1.

**TABLE 1:** Parameter estimates of the *in vitro* mathematical model

Parameter			Unit	Estimate (RSE(%)^*a*^)
Host growth				
$B_{0}$	Initial bacterial load		OD600	0.0163 (4.21)
$k_{growth}$	Bacterial growth rate constant		/h	0.854 (1.47)
$k_{death}$	Bacterial death rate constant		/h	0.0207 (8.23)
$k_{DP}$	Rate of conversion from dormant to proliferating subpopulations		/h	0.0307 (8.45)
$log_{10}\left(C\right)$	Proliferative capacity		$log_{10}CFU$	7.81 (0.186)
Phage-bacteria interaction				
$log_{10}\left(k_{inf,W}\right)$	Log_10_-transformed adsorption rate constant of the phage strains to wild type bacteria	MP-A	$log_{10}mL/CFU/h$	−7.79 (0.317)
		PP-A	$log_{10}mL/CFU/h$	−7.25 (0.353)
		PP-B	$log_{10}mL/CFU/h$	−8.06 (0.508)
$log_{10}\left(k_{inf,M}\right)$	Log_10_-transformed adsorption rate constant of the phage strains to mutant bacteria	MP-A	$log_{10}mL/CFU/h$	−9.46 (0.296)
		PP-A	$log_{10}mL/CFU/h$	−9.95 (0.939)
		PP-B	$log_{10}mL/CFU/h$	−9.71 (0.76)
b	Burst size of phage	MP-A		29.1 (6.08)
		PP-A		20.05 (4.11)
		PP-B		19.5 (9.15)
Subpopulation structure				
$\phi_{WWW}$	logit coefficients^*b*^			0 fixed
$\phi_{WWM}$				−4.9 (3.21)
$\phi_{WMW}$				−7.81 (2.03)
$\phi_{WMM}$				−7.98 (2.4)
$\phi_{MWW}$				−6.83 (1.59)
$\phi_{MWM}$				−7.31 (2.99)
$\phi_{MMW}$				−17.1 (2.76)
$\phi_{MMM}$				−16.3 (2.77)

Cross-resistance probability		
Fraction resistant	Estimated value	Expected value under independent mutations
MP-A	$1.73\times 10^{-3}$	–^*c*^
PP-A	$7.41\times 10^{-4}$	–^*c*^
PP-B	$8.37\times 10^{-3}$	–^*c*^
MP-A + PP-A	$1.20\times 10^{-7}$	$1.28\times 10^{-6}=\left(1.73\times 10^{-3}\right)\times \left(7.41\times 10^{-4}\right)$
MP-A + PP-B	$6.62\times 10^{-4}$	$1.45\times 10^{-5}=\left(1.73\times 10^{-3}\right)\times \left(8.37\times 10^{-3}\right)$
PP-A + PP-B	$3.39\times 10^{-4}$	$6.20\times 10^{-6}=\left(7.41\times 10^{-4}\right)\times \left(8.37\times 10^{-3}\right)$
MP-A + PP-A + PP-B	$8.25\times 10^{-8}$	$1.07\times 10^{-8}=(1.73\times 10^{-3})\times (7.41\times 10^{-4})\times (8.37\times 10^{-3})$

^*a*^ RSE(%): relative standard error.

^*b*^ W: wild type; M: mutant; $Fraction=\frac{exp\left(\phi_{xyz}\right)}{\sum_{i,j,k\in \left\{W,M\right\}}exp\left(\phi_{ijk}\right)}$; the digits *x*, *y*, and *z* in the subscript of $\phi_{xyz}$ correspond to MP-A, PP-A, and PP-B, respectively.

^*c*^ "–", not applicable.

### Parameter estimation

Estimated infectivity constants ($k_{inf,W}$) matched observed potency (PP-A > MP-A >PP-B). Inundation thresholds (ITs), calculated as bacterial growth rate divided by $k_{inf,W}$, were $1.5\times 10^{7}$ (PP-A), $5.3\times 10^{7}$ (MP-A), and $9.8\times 10^{7}$ PFU/mL (PP-B). For M variants, MP-A showed the highest infectivity, explaining its superior suppression of resistant regrowth (Fig. 1A).

Estimated resistance fractions were lowest for PP-A ($7.4\times 10^{-4}$), followed by MP-A and PP-B. The observed co-resistant fraction for MP-A + PP-A ($1.2\times 10^{-7}$) was 10-fold lower than expected under independent mutation, suggesting reduced cross-resistance. In contrast, co-resistance in MP-A +PP-B and PP-A + PP-B exceeded expectations by 10- to 100-fold, indicating cross-resistance.

### Non-monotonous dose-exposure relationship accounts for faster resistance development with higher dose

Simulations revealed a non-monotonous relationship between initial phage dose (MOI) and phage titer at 24 h (Fig. 4B and C); the overall dose-exposure relationship was U-shaped. The decreasing portion of the U-shape curve occurs because higher doses kill bacteria rapidly, limiting amplification of phage. The increasing portion of the U-shape curve occurs because phage amplification becomes negligible beyond a critical threshold and higher initial dose drives higher phage exposure.

The range tested in our *in vitro* kinetic assays corresponded to the decreasing portion of the U-shape curve. Because of decreasing phage exposure with increasing phage MOI, resistant bacteria likely grew faster with higher phage MOI in our *in vitro* kinetic assays (Fig. 1A, B and 4B).

### Mechanism of cocktail synergy and selection of cocktail composition

Simulations of two-phage cocktails ($10^{4}$ PFU/mL total) showed that the extent of early bacterial growth suppression (<6 h) aligns with that of the stronger phage, with negligible contribution from the weaker phage. Therefore, mono-phage treatment using the strongest phage (i.e., PP-A) is expected to yield the best outcome with regards to early bacterial killing. However, the benefit of the phage cocktail emerged when the stronger phage suppressed susceptible bacteria (“predator”), while the weaker phage successfully eliminated resistant subpopulations (“scavenger”) (Fig. 4D). Specifically, in MP-A + PP-A, PP-A-resistant bacteria emerged but were rapidly eliminated by MP-A, leading to a longer period of bacterial suppression. In MP-A + PP-B and PP-A + PP-B, a substantial proportion of resistant bacteria was co-resistant to PP-B (reflecting the high cross-resistance of PP-B with MP-A and PP-A), reducing the efficacy of phage cocktails compared to monotherapy.

Across all cocktail simulations, we observed early competition between phages, wherein the more virulent phage suppressed the initial expansion of the weaker phage (Fig. 4D). For example, MP-A proliferation was reduced when co-administered with PP-A, and PP-B growth was inhibited when combined with either MP-A or PP-A.

The simulation results suggest that cocktail synergy stems primarily from the predator-scavenger complementarity that critically depends on the extent of phage-phage cross-resistance. Among the phage candidates tested, the optimal two-phage cocktail was MP-A + PP-A, with PP-A acting as the predator and MP-A as the scavenger. MP-A’s role as a scavenger was maximized by MP-A and PP-A’s low cross-resistance. A three-phage cocktail of MP-A + PP-A + PP-B is expected to offer negligible contribution to overall efficacy due to PP-B’s high cross-resistance with both MP-A and PP-A.

### Validation of the *in vitro* mathematical model

The *in vitro* model generated several testable hypotheses regarding phage-bacteria dynamics under cocktail treatment. First, during the early treatment phase, phage-phage competition favors preferential amplification of the stronger “predator” phage. Second, sustained bacterial suppression requires the weaker “scavenger” phage to infect and replicate on bacteria that have acquired resistance to the predator phage. Third, eventual bacterial regrowth arises from the emergence of dual-resistant mutants rather than from loss of phages. Lastly, higher initial phage doses are predicted to reduce subsequent phage replication, leading to lower long-term phage titers.

**Fig 5:**
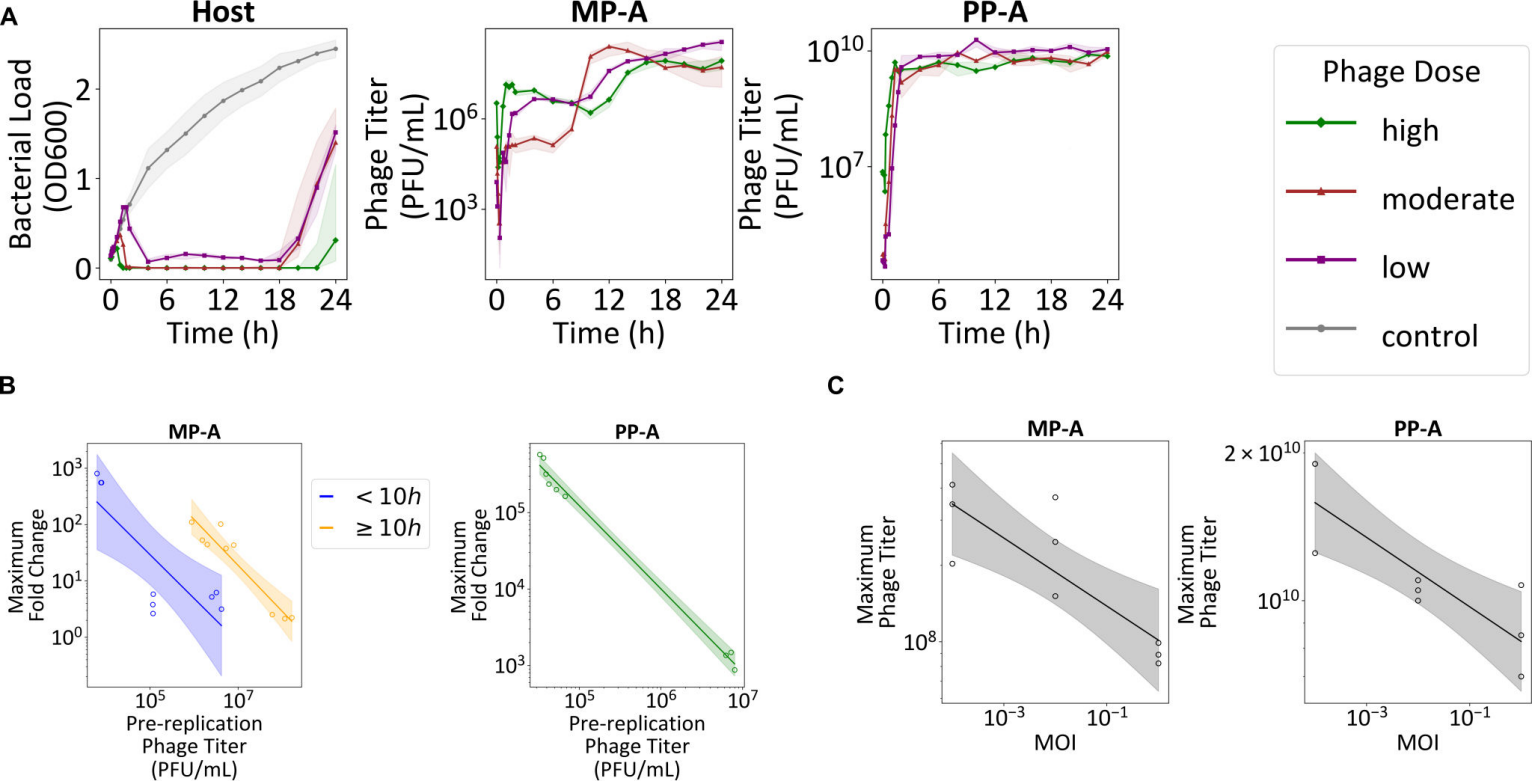
Experimental validation in MP-A + PP-B cocktail. (**A**) Bacterial and phage titers were quantified in an *in vitro* kinetic experiment involving the MP-A + PP-A cocktail. (**B**) The magnitude of phage proliferation exhibited an inverse relationship with the pre-replication phage titer. A linear regression line, along with its corresponding 95% confidence interval, is depicted based on log-transformed data. (**C**) The peak phage titer also demonstrated an inverse correlation with the dosage administered. A linear regression line, along with its 95% confidence interval based on log-transformed data, is presented.

To validate these aspects, we tracked *P. aeruginosa* 15-4 density and MP-A/PP-A titers over 24 h following MP-A + PP-A treatment (Fig. 5A). Early bacterial decline was accompanied by preferential proliferation of PP-A, supporting the first hypothesis. A transient resurgence at 8–12 h coincided with delayed MP-A amplification, followed by a second bacterial decline, consistent with the second hypothesis. Regrowth by 20 h likely reflected the emergence of dual-resistant bacteria, as both phage titers remained relatively stable rather than declining. Finally, the maximum fold change in phage titers was inversely correlated with both the pre-replication phage titer (Fig. 5B) and MOI (Fig. 5C), verifying the predicted dose-dependent suppression of long-term phage amplification.

### Model performance across 12 additional clinical isolates

To assess the generalizability of the model, we applied it to 12 additional *P. aeruginosa* strains with an initial bacterial inoculum of 10^8^ CFU/mL and phage doses spanning MOIs of 0.01, 1, and 100. Model parameters were estimated individually for each strain, and the resulting values are provided in Table S2. The initial conditions for model simulations were adjusted using a correction factor of 20 to match experimental bacterial density measurements. Simulated bacterial trajectories closely matched observed data across all strains (Fig. S3A through C), supporting the model’s robustness.

From the parameter estimates, we computed strain-specific resistance probabilities for MP-A, PP-A, and PP-B (Table S3). Using these values, we derived a synergy prediction criterion based on whether observed cross-resistance was lower than that expected under independence. Strains meeting this criterion were predicted to benefit from the MP-A + PP-B cocktail. Based on this analysis, only two out of 12 strains were predicted to benefit from the MP-A + PP-A cocktail, while the remaining strains were best treated with MP-A alone.

Among these, two strains exhibited complete suppression of bacterial load at 24 h following MP-A + PP-A treatment at MOI 100 (Fig. S3C). Notably, 50% of the strains satisfying the synergy criterion showed complete bacterial clearance, compared to only 10% of the strains that did not. While this criterion is not perfectly predictive, it effectively enriches strains more likely to exhibit strong synergy from combination therapy.

Adsorption rates and resistance probabilities showed substantial inter-strain variability. MP-A had higher average efficacy and lower resistance probabilities, while PP-A showed greater variability (Fig. S4). Hierarchical clustering of parameter profiles identified distinct strain groupings, suggesting differences in virulence or phage responsiveness (Fig. S5).

These results confirm the model’s generalizability and utility in characterizing strain-specific phage-bacteria dynamics.

### Model extension for *In Vivo* conditions

To simulate *in vivo* dynamics, we expanded the model to include phage PK and host immunity (Fig. 3D and E).

### Phage PK module

Phage disposition was modeled using a two-compartment structure comprising a central compartment $P_{C}$ and a peripheral compartment $P_{P}$. Phage elimination from the central compartment followed first-order kinetics with rate constant $k_{el}$, while distribution between compartments was governed by inter-compartmental transfer rates $k_{12}$ and $k_{21}$ are distribution rate constants between the central and peripheral compartments (Fig. 1C) The model equations are as follows:


(5) $$\begin{array}{rl} & \frac{d}{dt}P_{C}=(b-1)\cdot P_{C}\cdot \left(k_{\text{inf,W }}\cdot B_{W,P}+k_{\text{inf,M }}\cdot B_{M,P}\right)\\ & \;-k_{12}\cdot P_{C}+k_{21}\cdot P_{P}-k_{el}\cdot P_{C} \end{array}$$

(6) $$\begin{array}{c} \frac{d}{dt}P_{P}=k_{12}\cdot P_{C}-k_{21}\cdot P_{P} \end{array}.$$

### Immune and bacterial modules

Bacterial dynamics were modeled as *in vitro* but with several extensions. Dormant bacteria were assumed to be immune-evasive and phage-refractory(30). Most bacterial parameters were retained from the *in vitro* model, except for a few key updates to better capture *in vivo* dynamics: bacterial carrying capacity (set to $10^{8.79}$CFU/mL), PP-A burst size (adjusted to 170), and wild-type log_10_ PP-A infectivity constant (adjusted to −7.67). This simultaneous increase in burst size and decrease in adsorption rate reflects established inverse relationships between latent period and burst size (31), as longer latent periods (not explicitly modeled here) are indirectly captured through lower infectivity values. Phage clearance and transfer rates between lung compartments were calibrated to optimize fit to *in vivo* data. Model equations for a single phage are shown below. For full model equations for phage cocktails, refer to the Supplementary Methods.


(7) $$\begin{array}{c} \frac{d}{dt}B_{W,P} \end{array}=\begin{array}{l} k_{growth}\cdot B_{W,P}-P_{C}\cdot k_{inf,W}\cdot B_{W,P}-k_{PD}\cdot B_{W,P}+k_{DP}\cdot B_{W,D}\\ -k_{death}\cdot B_{W,P}-\epsilon \cdot I\cdot B_{W,P}\cdot \frac{1}{1+\frac{B_{P}}{B_{50}}} \end{array}$$

(8) $$\begin{array}{c} \frac{d}{dt}B_{M,P} \end{array}=\begin{array}{l} k_{growth}\cdot B_{M,P}-P_{C}\cdot k_{inf,M}\cdot B_{M,P}-k_{PD}\cdot B_{M,P}+k_{DP}\cdot B_{M,D}\\ -k_{death}\cdot B_{M,P}-\epsilon \cdot I\cdot B_{M,P}\cdot \frac{1}{1+\frac{B_{P}}{B_{50}}\;} \end{array}$$

(9) $$\begin{array}{c} \frac{d}{dt}B_{W,D}=k_{PD}\cdot B_{W,P}-k_{DP}\cdot B_{W,D}-k_{death}\cdot B_{W,D} \end{array}.$$

(10) $$\begin{array}{c} \frac{d}{dt}B_{M,D}=k_{PD}\cdot B_{M,P}-k_{DP}\cdot B_{M,D}-k_{death}\cdot B_{M,D} \end{array}.$$

(11) $$\begin{array}{c} \frac{d}{dt}I=\alpha \cdot B_{P}\cdot \frac{1}{1+\frac{B_{P}}{B_{50}\prime }}-\beta \cdot I \end{array}.$$

**Fig 6:**
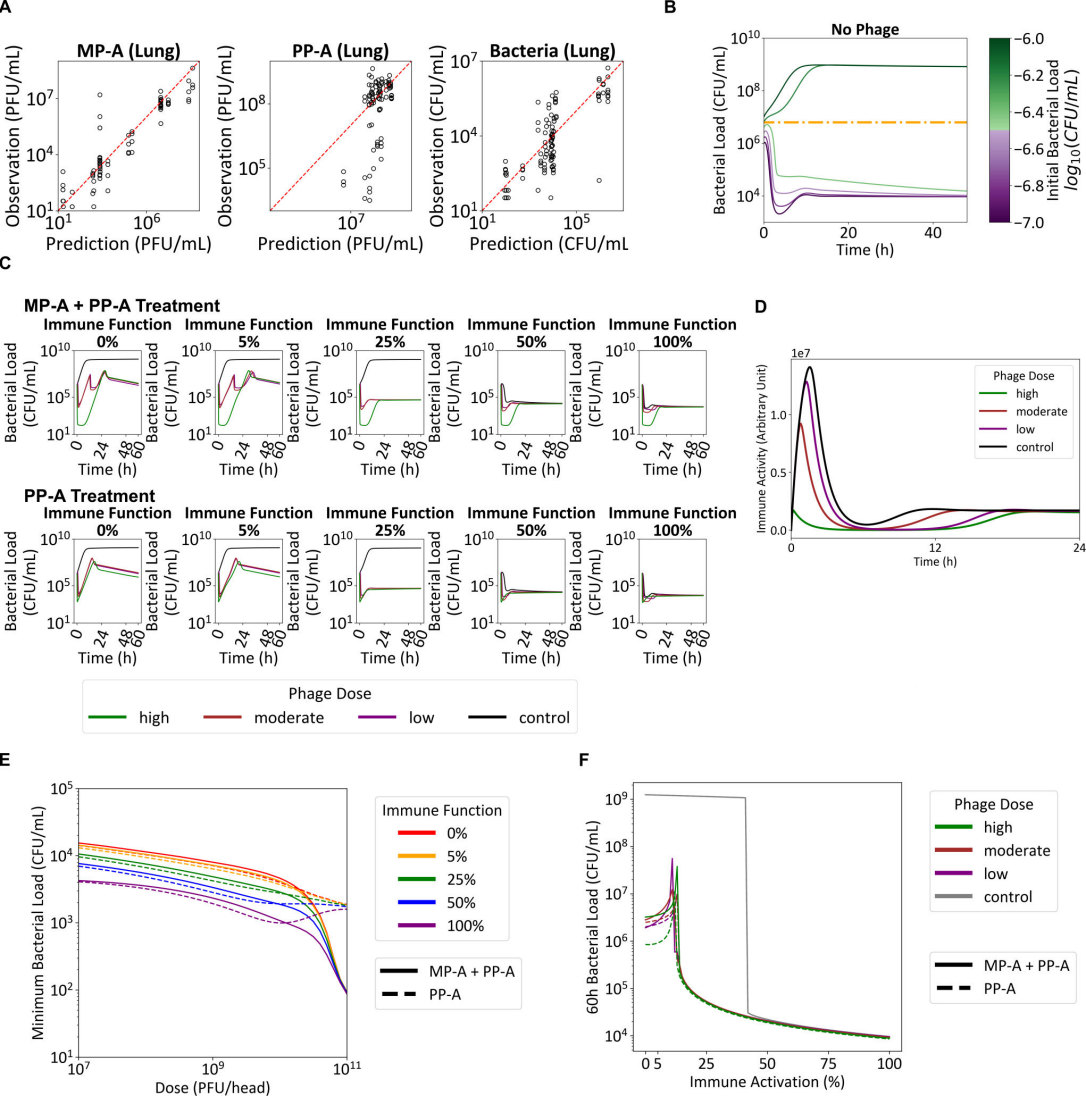
Extension and calibration of the *in vitro* bacteria-phage dynamics model to predict *in vivo* PKPD. (**A**) The goodness-of-fit plot comparing observed and predicted values is presented. Only the data points observed before the first mortality for each dosing group are shown. (**B**) Simulations examining the relationship between bacterial clearance and the initial bacterial load were conducted. (**C**) The temporal dynamics of bacterial clearance were analyzed for PP-A monotherapy and the combined MP-A + PP-A therapy, with outcomes dependent on varying levels of immune function. Simulations were performed across different phage dosage conditions, including control, low (10^7^ PFU/head), moderate (10^9^ PFU/head), and high (10^11^ PFU/head). (**D**) Simulations examining the time course of immune activity were conducted for varying phage doses, including control, low (10^7^ PFU/head), moderate (10^9^ PFU/head), and high (10^11^ PFU/head). (**E**) The minimum bacterial load in simulations with varying immune function levels (0, 5, 25, 50, and 100%) and doses (10^7^ to 10^11^ CFU/head). (**F**) The 60-h bacterial load in simulations with varying doses (control, low [10^7^ PFU/head], moderate [10^9^ PFU/head], and high [10^11^ PFU/head]) and immune function levels (0 to 100%) are shown.

The extended model provided a good fit to experimental *in vivo* data (Fig. 6A). In contrast, a reduced model excluding immune-mediated bacterial clearance consistently overestimated both phage titers and bacterial burden (Fig. S6), emphasizing the necessity of incorporating host immunity for accurate simulation.

### Simulating *in vivo* dynamics

The model exhibited bistability: bacterial loads were either controlled or reached maximal levels, depending on the initial inoculum (Fig. 6B). This threshold effect was driven by the saturable immune-mediated bacterial clearance.

Minimum bacterial load decreased with dose, similar to the tendency seen *in vitro*. However, long-term bacterial load showed a different tendency. Simulations of MP-A + PP-A and PP-A monotherapy across varying immune function levels revealed that long-term bacterial control was determined by immunity, not phage dose (Fig. 6C and F). With ≥25% immune function, bacterial levels converged to about $10^{4}$CFU/mL regardless of phage dose. At ≤5% immune function, bacterial outgrowth was predicted.

MP-A + PP-A showed limited benefit over PP-A at low doses but outperformed monotherapy at $\geq 10^{10}$ PFU (Fig. 6E) in reducing minimum bacterial load. This is because doses that yield exposures higher than the ITs can kill all bacteria except those co-resistant to MP-A and PP-A. Additional simulations indicated that high phage doses reduced immune activation (Fig. 6D), consistent with reports of attenuated inflammatory cytokine release at high phage exposure (15).

### *In vivo* model validation

#### Comparative efficacy of phage treatment

**Fig 7:**
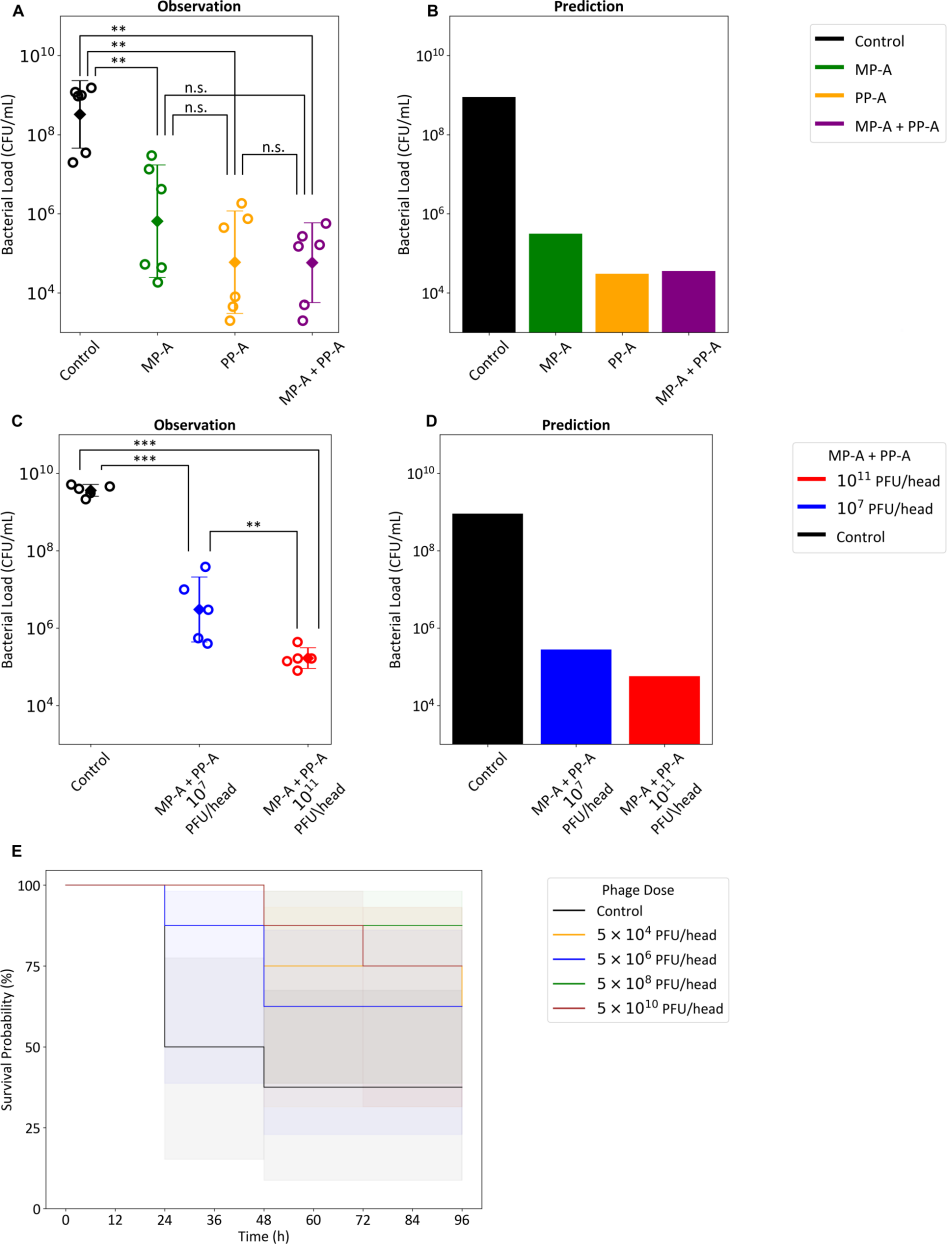
Validation of the *in vivo* phage-bacteria dynamics model. The model validation involved assessing the comparative *in vivo* efficacy of monotherapy and cocktail therapy (**A and B**) and dose-response relationships (**C and D**) in mice with acute pneumonia. **A** and **C** show the observed bacterial densities measured at 21 (**A**) and 20 (**C**) h post-infection, whereas (**B**) and (**D**) present the predicted bacterial density for the time points 21 and 20 h, respectively. In **A **and **C**, the error bars represent the mean ± standard deviation for the log_10_-transformed data. ANOVA indicated significant differences, which were further examined using Tukey’s HSD test. Statistical significance is denoted as follows: not significant (n.s.), **P < 0.01, ***P < 0.001. (**E**) The Kaplan-Meier plot shows the survival of mice for varying levels of MP-A + PP-A cocktail phage dose.

In a murine acute pneumonia model ($10^{7.7}$CFU/mouse), mice were treated with MP-A, PP-A, or the MP-A + PP-A cocktail ($5\times 10^{9}$ PFU/head, intravenous (i.v.). All treatments significantly reduced lung bacterial loads relative to control ($10^{8.52}$CFU/lung), with final counts of $10^{5.18}$ (MP-A), $10^{4.77}$ (PP-A), and $10^{4.78}$ (cocktail). However, no additional benefit was observed for the cocktail over PP-A alone (Fig. 7A).

Simulations assuming $10^{7.7}$CFU/mouse and a 1 mL lung volume accurately recapitulated these findings, including the lack of additive benefit from combination therapy at this dose (Fig. 7B).

### *In vivo* dose-response and survival outcomes

To assess model extrapolation, we conducted a dose-response study using a higher inoculum ($10^{7.78.17}$CFU/mouse). Lung bacterial loads at 20-h post-infection were $10^{9.56}$ (control), $10^{6.52}$ ($10^{7}$ PFU), and $10^{5.19}$ ($10^{11}$ PFU), with model predictions closely matching observed trends (Fig. 7C and D).

Survival outcomes also improved with dose. In a separate pneumonia model ($5\times 10^{8}$CFU/mouse), 3/8 mice survived in controls. Survival rates with phage doses of $5\times 10^{4}$, $10^{6}$, $10^{8}$, and $10^{10}$ PFU/head were 5/8, 5/8, 7/8, and 6/8, respectively (Fig. 7E), supporting a positive dose-response relationship.

## DISCUSSION

This study presents a translational PKPD modeling framework tailored to the unique biological properties of bacteriophage therapy. Building on classical predator-prey dynamics (32), we show that accurate interpretation of *in vitro* kinetic assays requires accounting for both phage resistance and bacterial dormancy. Our model suggests that phage-resistant mutants may retain partial susceptibility, explaining variable regrowth patterns across phages. Dormancy, modeled as a reversible, density-dependent transition to a phage-insensitive state, also contributed to incomplete suppression at low doses. Partitioning bacterial populations into combinatorial resistance variants enabled quantitative estimation of cross-resistance, informing cocktail design.

Previous modeling studies of phage therapy have ranged from empirical curve fitting to simple predator-prey frameworks. Most former research formalizing phage-bacteria dynamics largely omitted dormancy and partial resistance (33, 34). Other research exploring resistance evolution assumed fully binary phage-bacteria interactions (35). Previous modeling efforts have demonstrated immune-phage synergy experimentally coupled with dynamic modeling (14). One previous work introduced an approach of dividing the total bacterial population into two subpopulations: one growing drug-susceptible population and one resting insusceptible population (36). Our model unifies and extends these prior frameworks by incorporating density-dependent dormancy, partial resistance, phage competition, and host immunity—each calibrated against time-resolved experimental data. This positions our framework as a biologically grounded, pharmacologically actionable extension of existing models.

Model simulations revealed a distinctive non-monotonous dose-exposure relationship due to low doses allowing bacterial expansion and subsequent phage amplification and high doses rapidly eliminating bacteria and limiting replication. This non-linear dynamic, previously suggested in theoretical models (18), underscores the need to redefine phage dosing paradigms, as increasing dose may not linearly improve efficacy.

Our study also revealed the functional basis for cocktail synergy *in vitro*: the “predator-scavenger” dynamic. Here, one phage reduces the dominant population, while a second eliminates resistant survivors. This was evident in the MP-A + PP-A combination, where MP-A suppressed PP-A resistant variants. Cross-resistance is a pivotal factor determining cocktail complementarity since effective scavenging requires that bacteria resistant to the predator remain susceptible to the scavenger.

The model further highlights the critical role of host immunity in sustaining bacterial control. While high phage doses can accelerate bacterial clearance and potentially reduce immune activation, durable suppression requires sufficient immune function (14, 16, 37). In immunocompetent hosts, minimal cocktails may suffice because innate immunity clears residual phage-resistant populations and prevents bacterial regrowth. The benefit of phage cocktail primarily stems from the greater transient killing of bacteria at sufficiently high doses. In immunocompromised settings, however, the “predator-scavenger” synergy in phage cocktails becomes highly valid, and adjunctive strategies—such as combination with antibiotics or immunomodulation—may be necessary to further suppress resistance emergence. These findings support a tailored, host-contextual approach to phage therapy.

A key strength of our framework is its generalizability. Application to 12 additional *P. aeruginosa* clinical isolates confirmed the model’s robustness across phenotypic diversity. Despite variations in susceptibility, the model reliably captured killing kinetics, resistance dynamics, and synergy, supporting its potential utility in guiding strain-specific phage selection and dose optimization.

Several limitations warrant consideration. Our current modeling framework focuses on innate immune clearance of proliferating bacteria and does not explicitly incorporate adaptive immune mechanisms, such as anti-phage neutralizing antibodies, which can substantially alter phage PK and therapeutic efficacy in longer-term or repeated treatments. Future extensions of this model could integrate adaptive immunity components, leveraging recent studies that characterize neutralizing antibody kinetics in preclinical and clinical contexts (38, 39). Additionally, the model does not account for spatial heterogeneity, which could impact phage-bacteria dynamics in structured environments such as biofilms. *In vivo* validation was limited to acute lung infections in immunocompetent mice; future studies should extend to chronic infections and immunocompromised models. The contributions of dormancy in such contexts also merit further investigation.

In conclusion, this study presents a validated, mechanistic PKPD framework for bacteriophage therapy. By capturing resistance, non-monotonic dose-response, and host-pathogen interplay, our model supports rational dose selection and design of phage cocktails. It aligns with emerging priorities in model-informed drug development and offers a foundation for translating phage therapy into clinically effective strategies for MDR infections.

## Data Availability

All data analyzed and code used in this paper were deposited to Mendeley Data, which is freely accessible (https://doi.org/10.17632/w9kd3tnmf3.1). Any additional information required to reanalyze the data reported in this paper is available from the lead contact upon request, if permission is granted by Microbiotix.

## SUPPLEMENTAL MATERIAL

Supplemental Material msystems.01384-25-s0001.docx Supplemental methods, Figures S1 to S6, and Tables S1 to S6,

ASM does not own the copyrights to Supplemental Material that may be linked to, or accessed through, an article. The authors have granted ASM a non-exclusive, world-wide license to publish the Supplemental Material files. Please contact the corresponding author directly for reuse.
