# Supplementary material for: Model-informed development of bacteriophage therapy: bridging in vitro and in vivo efficacy against multidrug-resistant Pseudomonas aeruginosa
Source: mSystems. 2025 Nov 13;10(12):e01384-25. doi: 10.1128/msystems.01384-25 (PMC12710366; doi:10.1128/msystems.01384-25)
Supplement: Supplemental Material — Supplemental methods, Figures S1 to S6, and Tables S1 to S6, [file msystems.01384-25-s0001.docx]

Supplementary Material

# SUPPLEMENTARY METHODS

## Experimental model details

### Bacterial strains and phages

1) Phage isolation and preparation

Phages MP-A, PP-A, and PP-B were isolated from sewage samples collected at Severance Hospital, Seoul, Korea. Samples were treated with 1 M NaCl and refrigerated at 4°C for 24 hours. Following centrifugation at 8000 g for 30 minutes at 4°C (Supra R22; Hanil, Korea), the supernatant was filtered through a 0.22-$\mu$m pore-size membrane (Corning, NY, USA). Polyethylene glycol (PEG) 8000 at 10% concentration (Sigma, St. Louis, MO, USA) was added and the mixture was incubated for another 24 hours at 4°C. Phages were pelleted by centrifugation at 14000 g for 1 hour at 4°C, resuspended in sterilized SM buffer (100 mM NaCl, 8 mM MgSO4, 50 mM Tris-HCl, pH 7.5), and further sterilized by passage through a 0.22 $\mu$m syringe filter.

2) Host strain and maintenance

The *P. aeruginosa* 15-4 strain was isolated from clinical samples of a pneumonia patient from Severance Hospital. Its resistance to carbapenems (imipenem and meropenem) was confirmed using the VitekN132 system (bioMérieux, Marcy-l’Étoile, France) and CLSI disk diffusion method. The strain was maintained as a frozen stock in Luria broth LB medium with 25% glycerol at -70°C and reactivated for experiments by overnight incubation at 35°C in LB broth on a rotary shaker at 180 rpm.

3) Phage-sensitivity testing

Sensitivity of *P. aeruginosa* 15-4 to the phages was assessed using the spot test on Mueller-Hinton II agar (MHA) plates (Asanpharm, Seoul, Republic of Korea). The host bacteria were spread evenly on the agar plates; phages were spotted, followed by serial dilution and incubation using the double-layer agar method. Single-plaque phages were harvested, amplified in LB medium with the host strain, and cleared of cell debris by centrifugation and filtration. The phage preparations were then stored in 1.5-mL aliquots at 4°C.

### Animal model

The experimental protocols using mice were approved by the Institutional Animal Care and Use Committee of the respective institutions where the experiments were conducted (HDS Bio, Pohang-si, Republic of Korea, IACUC ID: 20231024-21; DT&CRO, Yongin-si, Republic of Korea, IACUC ID: 23E019; and HLB Biostep, Incheon, Republic of Korea, IACUC ID: 24-HB-0126).

Six-week-old male ICR mice were used for both the bacterial dose-ranging survival experiments and the *in vivo* PKPD studies. In the *in vivo* survival experiments evaluating dose-dependent responses to phage treatment, seven-week-old female ICR mice were employed. The mice were housed in polycarbonate cages, under a temperature of 20–26℃ and relative humidity of 40–70%, with free access to food (Rodent Diet, Purina®, Neenah, WI, USA).

For the *in vivo* lung bacterial load experiments, five-week-old male ICR mice were used to compare lung bacterial loads following MP-A and PP-A monotherapy with those after administration of the MP-A + PP-A cocktail, all administered at the same dose. To compare different doses of the MP-A + PP-A cocktail, six-week-old female mice were used. The mice were housed in polycarbonate cages, under a temperature of 19℃ and relative humidity of 50–60%, with free access to food (Teklad Certified Irradiated Global 18% Protein Rodent Diet 2918C, Envigo RMS, Inc., IN, USA).

$CO_{2}$ was used to euthanize all animals.

1) Preparation of *P. aeruginosa* for inoculation

The *P. aeruginosa* 15-4 strain was prepared using the following protocol. The bacteria stored at -80°C were smeared on MHA and incubated at 37°C for 24 hours. Two colonies were picked from the MHA, one for establishing the ratio between optical density (OD) and CFU and the other for inoculation into the mice. The colonies were each inoculated in the Mueller-Hinton II Broth (MHIIB). The inoculated broth was incubated in a shaking incubator at 150 rpm for 24 hours at 37°C. The media was centrifuged at 15,000 rpm (21206 x g), for 2 minutes. The pellet was resuspended in 1 ml of autoclaved normal saline solution. For the first colony, the resuspended bacterial solution was diluted by 10-fold and the OD was measured using a spectrophotometer (Epoch2, Biotek, Seoul, Korea). Subsequently, 100 $\mu$L of the mixture was smeared on MHA and incubated at 37°C overnight. The number of colonies was compared to the OD600 to calculate the concentration of bacteria in the mixture. For the second colony, based on the OD600 measured by the spectrophotometer, the mixture was diluted to the target levels to be used for inoculation into mice.

2) Inoculation of *P. aeruginosa* 15-4 into mice

A total of 20 $\mu$L *P. aeruginosa* 15-4 mixture prepared above was inoculated intranasally into the mice 1 or 2 hours before injection of the phage treatments into the tail vein.

## Method details

### Bacteriophage characterization

1) Genomics analysis

Genomic DNA was extracted from the phage stock using the Phage DNA Isolation Kit (Norgen Biotek, ON, Canada) and quantified using the QuantStudio 3 (Thermo Fisher Scientific, MA, USA) system. The prepared DNA was then used for library construction for whole-genome sequencing (WGS) using the TruSeq Nano DNA Kit (Illumina, CA, USA). Sequencing was performed on an Illumina sequencing platform based on Sequencing by Synthesis (SBS) technology.

Raw sequencing reads were processed to remove adapters and low-quality bases using Trimmomatic v0.36.(1) Quality-filtered reads were assembled de novo using SPAdes v3.15.0.(2) The resulting contigs were annotated using Prokka v1.14.6 and compared to reference databases (NCBI BLAST) to identify homologous sequences.(3) Genome coverage and completeness were assessed using QUAST v5.0.2.(4) Phage genomes were visualized and analyzed for key genes related to phage structure, replication, and host interaction. Annotation of tRNAs was performed using tRNAscan-SE v2.0.7.(5)

2) Electron microscopy

Phage morphology was visualized using transmission electron microscopy (TEM). Purified phage suspensions were deposited onto Formvar/carbon-coated 200 mesh copper grids (TED PELLA, INC., CA, USA) and allowed to adsorb for 15 seconds. Excess liquid was removed with 110 mm filter paper (ADVENTEC, Tokyo, Japan), and the grids were negatively stained with 1% (w/v) uranyl acetate for 30 seconds. The grids were air-dried and examined using HT7800 TEM (HITACHI, Ltd., Tokyo, Japan) at the Yonsei Biomedical Research Institute, Yonsei University college of medicine.

Micrographs were captured using OneView 4K digital camera (Gatan, Inc., CA, USA). Morphological features, including head diameter, tail length, and tail type (contractile or non-contractile), were measured from the images using ImageJ software v1.53t.(6) Phages were classified according to the International Committee on Taxonomy of Viruses (ICTV) guidelines into the appropriate families (e.g., Myoviridae, Podoviridae).

3) Phage adsorption assay

A single colony of host bacteria (MXB1001, PAE 15-4) was cultured in 5 mL of LB medium at 35°C with shaking (180 rpm) for 16 hours. This pre-culture was then expanded into 20 mL of LB medium until reaching an OD600 of 0.5. The bacterial cells were mixed with phages MP-A, PP-A, or PP-B with MOI of 10-3, vortexed, and filtered through a 0.22-$\mu$m pore-size syringe filter. Samples (1 mL) were collected at intervals from 0 to 30 minutes and analyzed for phage concentration using the double agar overlay method. The experiments were performed in triplicate.

4) One-step growth assay

Similar to the adsorption assay, a single colony of MXB1001 (PAE 15-4) was incubated in 5 mL of LB medium at 35°C with shaking (180 rpm) for 16 hours. Subsequently, 500 $\mu$L of this culture was transferred into three 5 mL tubes of LB medium and incubated until an OD600 of 0.2 was reached.

Bacteria were mixed with phages with MOI of 10-3 for MP-A and 10-5 for PP-A and PP-B. The mixture was incubated for 10 minutes at 18–29°C and then centrifuged at 10,000 rpm (9,400 x g) for 10 minutes; the supernatant was discarded, and pellets were resuspended in 1 mL of LB medium. This washing step was repeated once. Twenty microliters from the final suspension were inoculated into 20 mL of LB medium and incubated at 37°C with shaking (180 rpm). Samples (100 $\mu$L) were taken every 5 minutes for 2 hours and processed using the double agar overlay method to quantify phages. The experiments were performed in triplicate.

### *In vitro* bacterial kinetic assay

1) Conventional high-throughput assay

(i) Preparation: A single colony of host bacteria (MXB1001, PAE 15-4) was cultured in 5 mL of MHIIB in a 15-mL conical tube and incubated at 35°C with shaking (180 rpm) for 16 hours.

(ii) Inoculation: 100 $\mu$L of this culture was transferred into 9.9 mL of MHIIB in a 50-mL conical tube and incubated until it reached an OD600 of 0.12 (equivalent to approximately $1.00\times{10}^{8}$ CFU/mL).

(iii) Assay Setup: The assay was set up in a 96-well plate with a total volume of 100 $\mu$L per well. Bacteria were added to each well to achieve a starting concentration of $5.0\times{10}^{6}$ CFU/mL). Phage treatments (both single and cocktail) were added at 50 $\mu$L per well to achieve designated MOI values (ranging from 10-6 to 102); replicate numbers were recorded and are provided in Table S4.

(iv) Incubation and Measurement: Plates were incubated at 35°C for 24 hours in a Spectramax ABS spectrophotometer (Molecular Devices, San Jose CA, USA), with OD600 measurements taken hourly after shaking the plate.

2) Extended assay

This extended assay simultaneously measured bacterial load and phage titer.

(i) Preparation: A single colony was cultured in 10 mL of LB medium in a 15-mL conical tube at 35°C with shaking (180 rpm) until an OD600 of 0.2 was reached.

(ii) Dilution and Inoculation: 2 mL of this culture was diluted into 20 mL of LB medium. Phages were added to achieve the specified MOI. The control group consisted of two replicates. There were three treatment groups for which the MOI was 10-4, 10-2, and 100. Six replicates were conducted for each treatment group.

(iii) Incubation and Sampling: Single-phage treatments were incubated for six hours, with OD600 measurements taken hourly. The MP-A + PP-A cocktail was incubated for 24 hours, with hourly measurements of both OD600 and phage titers. Phage titers for MP-A were measured using PAE 14-4 (resistant to PP-A) and for PP-A using PAE 51540 (resistant to MP-A), employing the double agar overlay method. Conversion factors of 1/0.9 for MP-A and 1/0.4 for PP-A were applied to adjust plaque counts to levels equivalent to those obtained with PAE 15-4.

### Dormant bacteria formation assay

Host bacteria preserved in 20% glycerol stock were streaked onto Mueller-Hinton agar (MHA) plates and incubated at 35°C for 16–18 hours to facilitate colony formation. A single colony was then selected and inoculated into 5 mL of Mueller-Hinton broth (MHB), followed by incubation at 35°C with shaking at 180 rpm for 16–18 hours. The resulting culture was subsequently diluted 1:100 in fresh MHB and incubated under the same conditions until it reached a turbidity equivalent to the 0.5 McFarland standard. The bacterial culture was then dispensed into the wells of a 96-well plate, with nine wells containing a redox dye (Biolog, Inc., Hayward, CA, USA) and nine wells without the dye. For wells containing the redox dye, the culture-to-dye volume ratio was maintained at 9:1. A sealing film was applied to the 96-well plate, and optical density at 590 nm (OD590) was recorded at 20-minute intervals for a total duration of 1,420 minutes using the Odin spectrophotometer (Biolog, Inc., Hayward, CA, USA).

### Dormant bacteria kinetic assay

Host bacteria preserved in 20% glycerol stock were streaked onto a Mueller-Hinton agar (MHA) plate and incubated at 35°C for 16–18 hours to facilitate colony formation. A single colony was subsequently selected and inoculated into 5 mL of M9 medium, followed by incubation at 35°C with shaking at 180 rpm for 24 hours. The culture was then diluted 1:100 in fresh M9 medium and incubated at 35°C with shaking at 180 rpm for an additional 48 hours. Phages, diluted in phosphate-buffered saline (PBS), were added to achieve the MOI of 0.001 for the phage treated groups. Samples were collected at 24-hour intervals from the time of phage inoculation up to seven days post-inoculation to quantify bacterial and phage concentrations. To determine phage titer, 1 mL of each collected sample was filtered through a 0.22 $\mu$m membrane to remove bacterial cells. The filtrate was subsequently diluted in SM buffer and plated for plaque-forming unit (PFU) enumeration. To assess bacterial concentration, the bacterial sample was centrifuged at 10,000 $\times$ g for 5 minutes, after which the supernatant was carefully removed. The resulting bacterial pellet was resuspended in fresh PBS, serially diluted, and plated to determine viable bacterial counts. The dormant bacteria kinetic assay was conducted for three experimental groups: the control group, the MP-A treatment group, and the PP-A treatment group.

### Assessing the impact of bacterial dose on mice survival

Intranasal inoculation of mice with bacterial doses of ${10}^{6}$, ${10}^{7}$, $5\times{10}^{7}$ CFU/head was performed to establish an acute pneumonia mouse model. Survival assessments were conducted at six-hour intervals. Each treatment group consisted of 10 replicates.

### *In vivo* mouse PKPD experiment

The pharmacokinetics (PK) of the MP-A + PP-A cocktail in the noninfected mouse model and the pharmacokinetics-pharmacodynamics (PKPD) of the MP-A + PP-A cocktail in the infected mouse model were analyzed. The PKPD experiment in noninfected mice included a control group (three mice per timepoint) and three MP-A + PP-A treatment groups (three mice per timepoint), which were administered doses of ${10}^{7}$ PFU/head, ${10}^{9}$ PFU/head, and ${10}^{11}$ PFU/head, respectively. Similarly, the PKPD experiment in infected mice comprised a control group (four mice per timepoint) and three MP-A + PP-A treatment groups (four mice per timepoint), receiving doses of ${10}^{7}$ PFU/head, ${10}^{9}$ PFU/head, and ${10}^{11}$ PFU/head, respectively.

For the uninfected mouse model, the animals were fixed using a frame and using a 26-gauge needle, the control normal saline or the bacteriophage cocktail mixture was injected into the tail vein for one minute. The animals were sacrificed at 0, 0.5, 1, 4, 8, 12, 24, 48, and 60 hours after placebo or bacteriophage treatment.

For the infected mouse model, the bacterial mixture was prepared at a concentration of ${10}^{7}$ CFU/ 20 $\mu$L. The mice were fixed using the cervical skin fixation method, and the bacterial mixture was inoculated intranasally using a micropipette, 10 µL into each of the two nostrils. One hour after the infection, the animals were fixed using a frame and using a 26-gauge needle, the control normal saline or the bacteriophage cocktail mixture was injected into the tail vein for one minute. The animals were sacrificed at 0, 0.5, 1, 4, 8, 12, 24, 48, and 60 hours after placebo or bacteriophage treatment.

The animals to be sacrificed were anesthetized and the abdominal aorta was exposed using laparotomy. The blood was sampled from the abdominal aorta using a syringe and stored in a vacutainer tube with a clot activator. The blood sample was left for 15–20 minutes in room temperature for clotting. The clotted sample was centrifuged at 5000 rpm (7069 $\times$ g) for 5 minutes to extract the serum. After collection of serum, the animal was euthanized using CO${}_{2}$ and the lungs were extracted. The extracted lungs were measured for weight and were then homogenized using a bead homogenizer. The serum samples and homogenized lung samples were diluted and quantitated for bacterial load and phage titer.

### Phage pharmacokinetics analysis

The initial concentration $C_{0}$, area under the curve until the last timepoint $AUC_{last}$, terminal half-life, initial concentration normalized by dose (${C_{0}}/{Dose}$), and area under the curve until the last timepoint normalized by dose ($AU{C_{last}}/{Dose}$) were computed to characterize the pharmacokinetics of MP-A and PP-A in serum and lung tissue in both noninfected and infected mouse models. Phoenix® WinNonlin® software (Version 8.3, Certara, Radnor, PA, USA) was utilized for the calculation of these pharmacokinetic parameters, employing the linear trapezoidal method for $AUC_{last}$ determination.

### *In vitro* phage-bacteria dynamics mathematical model

In the development of the population dynamics model, we employed a deterministic approach using ordinary differential equations (ODEs). We followed the standard mass action kinetics framework, assuming that phages and bacteria are evenly distributed within a well-mixed environment and their concentrations can be approximated using a continuous scale.

$k_{growth}$​ is the bacterial growth rate constant, $k_{inf,W}$ and $k_{inf,M}$​ are the phage infectivity (or apparent adsorption rate) constants for wild-type and mutant bacteria, respectively, $k_{PD}$​ and $k_{DP}$​ describe transitions between proliferating and dormant states, and $k_{death}$​ represents basal bacterial death.

Dormancy was modeled as a density-dependent phenomenon: $k_{PD}={B_{tot}}/C$ where C is the carrying capacity and $B_{tot}$​ the total bacterial burden. To implement phage cocktail treatment, the above equations were extended to incorporate multiple phages and bacterial subpopulations with all possible resistance combinations to the administered phages (Fig.1C).

The following equations describe the dynamics of wild-type, proliferating bacteria ($B_{W,P}$), wild-type, dormant bacteria ($B_{W,D}$), mutant, proliferating bacteria ($B_{M,P}$), and mutant, dormant bacteria ($B_{M,D}$) against a single phage (P) (Fig.1B).

1) Wild-type bacteria

$$\begin{aligned} \frac{d}{dt}B_{W,P}=k_{growth}\cdot B_{W,P}-k_{inf,W}\cdot B_{W,P}-k_{PD}\cdot B_{W,P}+k_{DP}\cdot B_{W,D}-k_{death}\cdot B_{W,P} \\ \left( EquationS1 \right) \end{aligned}$$

$$\begin{aligned} \frac{d}{dt}B_{W,D}=k_{PD}\cdot B_{W,P}-k_{DP}\cdot B_{W,D}-k_{death}\cdot B_{W,D} \\ \left( EquationS2 \right) \end{aligned}$$

2) Resistant mutants

$$\begin{aligned} \frac{d}{dt}B_{M,P}=k_{growth}\cdot B_{M,P}-k_{inf,M}\cdot B_{M,P}\cdot P-k_{PD}\cdot B_{M,P}+k_{DP}\cdot B_{M,D}-k_{death}\cdot B_{M,P} \\ \left( EquationS3 \right) \end{aligned}$$

$$\begin{aligned} \frac{d}{dt}B_{M,D}=k_{PD}\cdot B_{M,P}-k_{DP}\cdot B_{M,D}-k_{death}\cdot B_{M,D} \\ \left( EquationS4 \right) \end{aligned}$$

3) Density dependence of dormant state transition

$$\begin{aligned} k_{PD}=\frac{B_{tot}}{C} \\ \left( EquationS5 \right) \end{aligned}$$

$$\begin{aligned} B_{tot}=B_{W,P}+B_{W,D}+B_{M,P}+B_{M,D} \\ \left( EquationS6 \right) \end{aligned}$$

4) Phages

$$\begin{aligned} \frac{d}{dt}P=\left( b-1 \right)\cdot\left( k_{inf,W}\cdot B_{W,P}\cdot P+k_{inf,M}\cdot B_{M,P}\cdot P \right) \\ \left( EquationS7 \right) \end{aligned}$$

($B_{W,P}$: Wild-type, proliferating bacteria, $B_{W,D}$: Wild-type, dormant bacteria, $B_{M,P}$: Mutant, proliferative bacteria, $B_{M,D}$:Mutant, dormant bacteria, $P$:Phages)

The initial condition of the system was as follows:

$$\begin{aligned} B_{W,P}\left( 0 \right)=B_{0}\cdot\frac{1}{1+exp\left( \phi_{M} \right)} \\ \left( EquationS8 \right) \end{aligned}$$

$$\begin{aligned} B_{M,P}\left( 0 \right)=B_{0}\cdot\frac{exp\left( \phi_{M} \right)}{1+exp\left( \phi_{M} \right)} \\ \left( EquationS9 \right) \end{aligned}$$

$$\begin{aligned} B_{W,D}\left( 0 \right)=B_{M,D}\left( 0 \right)=0 \\ \left( EquationS10 \right) \end{aligned}$$

$$\begin{aligned} P\left( 0 \right)=P_{0} \\ \left( EquationS11 \right) \end{aligned}$$

To extend the model to cocktail therapy, all possible phage-sensitivity combinations with regards to MP-A, PP-A, and PP-B were considered. A three-digit subscript notation, $B_{ijk}$ with $i,j,k\in\{W,M\}$, was used to represent each bacterial subpopulation. The proliferating or dormant state was additionally distinguished using an additionally indicator symbol of either P or D following a comma separator. Thus, the total bacterial concentration is a sum of all possible genetic variants in both P and D states:

$$\begin{aligned} B_{tot}=\sum_{i,j,k\in\left\{ W,M \right\}} B_{ijk,P}+\sum_{i,j,k\in\left\{ W,M \right\}} B_{ijk,D} \\ \left( EquationS12 \right) \end{aligned}$$

To reduce unnecessary clutter in the parameter symbols, we assigned integer identifiers of 1, 2, and 3 to phages MP-A, PP-A, and PP-B, respectively. Accordingly, the infection rate constants for MP-A, PP-A, and PP-B of the strain $B_{ijk,P}$ ($i,j,k\in\{W,M\}$) are denoted as $k_{inf,1}\left( ijk \right)$, $k_{inf,2}\left( ijk \right)$, and $k_{inf,3}\left( ijk \right)$, respectively. Hence, for $\theta\in\{1,2,3\}$,

$$\begin{aligned} k_{inf,\theta}\left( ijk \right)=\left\{ \begin{matrix} k_{inf,\theta,W} & ifi=W \\ k_{inf,\theta,M} & ifi=M \end{matrix} \right. \\ \left( EquationS13 \right) \end{aligned}$$

The following equations describe the dynamics of different bacterial variants, where $i,j,k\in\{W,M\}$,

$$\begin{aligned} \frac{d}{dt}B_{ijk,P}=k_{growth}\cdot B_{ijk,P}- \\ \end{aligned}$$

$$\begin{aligned} \frac{d}{dt}B_{ijk,D}=k_{PD}\cdot B_{ijk,P}-k_{DP}\cdot B_{ijk,D}-k_{death}\cdot B_{ijk,D} \\ \left( EquationS15 \right) \end{aligned}$$

The dynamics of phages are described by the following equations, where $\theta\in\{1,2,3\}$.

$$\begin{aligned} \frac{d}{dt}P_{\theta}=\left( b_{\theta}-1 \right)\cdot P_{\theta}\cdot\sum_{i,j,k\in\left\{ W,M \right\}} \left( k_{ijk}\cdot B_{ijk,P} \right) \\ \left( EquationS16 \right) \end{aligned}$$

The initial conditions were:

$$\begin{aligned} \phi_{WWW}=0 \\ \left( EquationS17 \right) \end{aligned}$$

$$\begin{aligned} B_{ijk,P}\left( 0 \right)=B_{0}\cdot\frac{exp\left( \phi_{ijk} \right)}{\sum_{x,y,z\in\left\{ W,M \right\}} exp\left( \phi_{xyz} \right)} \\ \left( EquationS18 \right) \end{aligned}$$

$$\begin{aligned} B_{ijk,D}\left( 0 \right)=0 \\ \left( EquationS19 \right) \end{aligned}$$

$$\begin{aligned} P_{\theta}\left( 0 \right)=P_{\theta,0} \\ \left( EquationS20 \right) \end{aligned}$$

The OD_600_ values are predicted by dividing B_tot_ by the factor $2\times{10}^{8}{OD_{600}\cdot mL}/{CFU}:$

$$\begin{aligned} y_{prediction}=\frac{B_{tot}}{2\times{10}^{8}} \\ \left( EquationS21 \right) \end{aligned}$$

### *In vitro* mathematical model parameter estimation

Monolix v2023R1 nonlinear mixed-effects modeling software (Lixoft, Antony, France) utilizing the SAEM algorithm was used to extend the model constructed above into a population model and estimate the parameter values. Variability between the 96-well plates and intra-plate variability were added to the parameters by assuming that each parameter followed a lognormal distribution. Residual variability was accounted for by using a combined proportional and additive model. The Monolix model file and the ‘.mlxtran’ file are deposited in the Mendeley Data repository.

For the parameters $B_{0}$, $k_{growth}$, $k_{death}$, $k_{DP}$, $b_{\theta}$, $k_{inf,\theta,W}$, and $k_{inf,\theta,M}$, lognormal distributions were assumed for the individual parameters:

$$\begin{aligned} log\left( \theta_{individual} \right)=log\left( \theta_{typical} \right)+\eta_{\theta}\left( \eta_{\theta}\sim N\left( 0,\omega_{\theta}^{2} \right) \right) \\ \left( EquationS22 \right) \end{aligned}$$

For the parameters $log_{10}C$, $\phi_{WWW}$, $\phi_{WWM}$, $\phi_{WMW}$, $\phi_{WMM}$, $\phi_{MWW}$, $\phi_{MWM}$, $\phi_{MMW}$, and $\phi_{MMM}$, a normal distribution was assumed for the individual parameters:

$$\begin{aligned} \theta_{individual}=\theta_{typical}+\eta_{\theta}\left( \eta_{\theta}\sim N\left( 0,\omega_{\theta}^{2} \right) \right) \\ \left( EquationS23 \right) \end{aligned}$$

Correlations were assumed for the inter-plate variability of $B_{0}$, $k_{growth}$, $k_{death}$, and $k_{DP}$:

$$\begin{aligned} \left[ \begin{matrix} \eta_{B_{0}} \\ \eta_{k_{growth}} \\ \eta_{k_{death}} \\ \eta_{k_{DP}} \end{matrix} \right] \\ \sim N\left( \left[ \begin{matrix} 0 \\ 0 \\ 0 \\ 0 \end{matrix} \right],\left[ \begin{matrix} \omega_{B_{0}}^{2} & cov_{k_{growth},B_{0}} & cov_{k_{death},B_{0}} & cov_{k_{DP,B_{0}}} \\ cov_{k_{g}rowth,B_{0}} & \omega_{k_{growth}}^{2} & cov_{k_{death},k_{growth}} & cov_{k_{DP},k_{growth}} \\ cov_{k_{death},B_{0}} & cov_{k_{death},k_{growth}} & \omega_{k_{death}}^{2} & cov_{k_{DP},k_{death}} \\ cov_{k_{DP},B_{0}} & cov_{k_{DP},k_{growth}} & cov_{k_{DP},k_{death}} & \omega_{k_{DP}}^{2} \end{matrix} \right] \right) \\ \left( EquationS24 \right) \end{aligned}$$

The residual variability was introduced using an additive error model:

$$\begin{aligned} y_{observation}=y_{prediction}+err_{a}\cdot\varepsilon\left( \varepsilon\sim N\left( 0,1 \right) \right) \\ \left( EquationS25 \right) \end{aligned}$$

### Application of the *in vitro* mathematical model to kinetic assays on 12 additional strains

The bacteria-phage dynamics model was applied to kinetic assay data from 12 additional *P. aeruginosa* strains (24_4, 24_11, 24_13, 24_17, 24_19, 24_22, 24_23, 24_32, 24_33, 24_37, 24_40, and 24_44) isolated from clinical samples. Kinetic assays were performed under four treatment conditions—Control, MP-A, PP-A, and MP-A + PP-A—with multiplicities of infection (MOIs) of 0.01, 1, and 100 applied to the phage treatment groups. Each treatment-MOI combination was conducted in triplicate. The model applied to these strains was structurally consistent with that used for *P. aeruginosa* strain 15-4, with modifications to parametrization methods, inclusion of baseline OD600 as an estimated parameter, and usage of a proportional error model.

A two-digit subscript notation, $B_{ij}$ with $i,j\in\{W,M\}$, was used to represent each bacterial subpopulation with i corresponding to resistance to MP-A and j corresponding to resistance to PP-A. The proliferating or dormant state was additionally distinguished using an additionally indicator symbol of either P or D following a comma separator. Thus, the total bacterial concentration is a sum of all possible genetic variants in both P and D states:

$$\begin{aligned} B_{tot}=\sum_{i,j\in\left\{ W,M \right\}} B_{ij,P}+\sum_{i,j\in\left\{ W,M \right\}} B_{ij,D} \\ \left( EquationS26 \right) \end{aligned}$$

To reduce unnecessary clutter in the parameter symbols, we assigned integer identifiers of 1, and 2 to phages MP-A and PP-A, respectively. Accordingly, the infection rate constants for MP-A and PP-A of the strain $B_{ij,P}$ ($i,j\in\{W,M\}$) are denoted as $k_{inf,1}\left( ij \right)$ and $k_{inf,2}\left( ij \right)$, respectively. Hence, for $\theta\in\{1,2\}$,

$$\begin{aligned} k_{inf,\theta}\left( ij \right)=\left\{ \begin{matrix} k_{inf,\theta,W} & ifi=W \\ k_{inf,\theta,M} & ifi=M \end{matrix} \right. \\ \left( EquationS27 \right) \end{aligned}$$

The following equations describe the dynamics of different bacterial variants, where $i,j\in\{W,M\}$,

$$\begin{aligned} \frac{d}{dt}B_{ij,P}=k_{growth}\cdot B_{ij,P}- \\ \end{aligned}$$

$$\begin{aligned} \frac{d}{dt}B_{ij,D}=k_{PD}\cdot B_{ij,P}-k_{DP}\cdot B_{ij,D}-k_{death}\cdot B_{ij,D} \\ \left( EquationS29 \right) \end{aligned}$$

The dynamics of phages are described by the following equations, where $\theta\in\{1,2\}$.

$$\begin{aligned} \frac{d}{dt}P_{\theta}=\left( b_{\theta}-1 \right)\cdot P_{\theta}\cdot\sum_{i,j\in\left\{ W,M \right\}} \left( k_{ij}\cdot B_{ij,P} \right) \\ \left( EquationS30 \right) \end{aligned}$$

The initial conditions were:

$$\begin{aligned} {fr}_{M\circ}=\frac{1}{1+exp\left( -\psi_{MP-A} \right)} \\ \left( EquationS31 \right) \end{aligned}$$

$$\begin{aligned} {fr}_{\circ M}=\frac{1}{1+exp\left( -\psi_{PP-A} \right)} \\ \left( EquationS32 \right) \end{aligned}$$

$$\begin{aligned} {fr}_{MM}=\zeta\cdot min\left( fr_{M\circ},fr_{\circ M} \right) \\ \left( EquationS33 \right) \end{aligned}$$

$$\begin{aligned} {fr}_{MW}=fr_{M\circ}-fr_{MM} \\ \left( EquationS34 \right) \end{aligned}$$

$$\begin{aligned} {fr}_{WM}=fr_{\circ M}-fr_{MM} \\ \left( EquationS35 \right) \end{aligned}$$

$$\begin{aligned} {fr}_{WW}=1-fr_{WM}-fr_{MW}-fr_{MM} \\ \left( EquationS36 \right) \end{aligned}$$

For, $i,j\in\{W,M\}$,

$$\begin{aligned} B_{ij,P}\left( 0 \right)=\frac{B_{0}}{20}\cdot fr_{ij} \\ \left( EquationS37 \right) \end{aligned}$$

$$\begin{aligned} B_{ij,D}\left( 0 \right)=0 \\ \left( EquationS38 \right) \end{aligned}$$

Here, 20 is a correction factor to relate the bacteria and phage nominal dose to the actual dose. The factor was calibrated to give good fit of the model.

For $\theta\in\{1,2\}$,

$$\begin{aligned} P_{\theta}\left( 0 \right)=\frac{P_{\theta,0}}{20} \\ \left( EquationS39 \right) \end{aligned}$$

Baseline ${OD}_{600,baseline}$ is added to the total bacteria transformed to OD600 using the factor $2\times{10}^{8}{OD_{600}\cdot mL}/{CFU}$.

$$\begin{aligned} y_{prediction}=\frac{B_{tot}}{2\times{10}^{8}}+{OD}_{600,baseline} \\ \left( EquationS40 \right) \end{aligned}$$

Model fitting followed a sequential approach: first estimating the parameters of the bacterial growth model, followed by the single-phage treatment model, and finally the phage cocktail treatment model. Principal component analysis (PCA) and clustering were then performed using the mean individual parameter estimates for each strain.

### The *in vivo* mathematical model

The models developed and fitted to *in vitro* kinetic curves were extended to incorporate *in vivo* phage pharmacokinetics and immune system dynamics.

Phage distribution was described using two compartments and phage elimination was assumed to occur in a linear fashion with elimination rate constant $k_{el,\theta}$ in the central compartment. $k_{12}$ and $k_{21}$ are distribution rate constants between the central and peripheral compartments.

Host immunity was modeled as being induced by the total population of proliferative (i.e. non-dormant) bacteria with an induction rate constant $\alpha$. The activation of the immune response was subject to density-dependent suppression by the bacterial load, where $B_{50}'$ represents the bacterial density at which the rate of immune system induction is reduced by half. Once activated, the immune system decays at a rate governed by the decay rate constant $\beta$.

Immune-mediated bacterial killing was also modeled as a saturable process, with an efficacy governed by the killing rate constant $\epsilon$. Saturation occurs as the proliferative bacterial population increases, with $B_{50}$ indicating the bacterial density at which immune killing is reduced to half its maximum rate.

Dormant bacteria were assumed to evade immune recognition and were therefore not subject to immune killing. Dose-to-lung concentration scaling was estimated by performing a regression analysis on dose-normalized initial lung concentrations.

The following system of ODE describes the updated model, where $\theta\in\{1,2,3\}$ and $i,j,k\in\{W,M\}$.

$$\begin{aligned} k_{PD}=\frac{B_{tot}}{C} \\ \left( EquationS41 \right) \end{aligned}$$

$$\begin{aligned} B_{tot}=\sum_{i,j,k\in\left\{ W,M \right\}} B_{ijk,P}+\sum_{i,j,k\in\left\{ W,M \right\}} B_{ijk,D} \\ \left( EquationS42 \right) \end{aligned}$$

$$\begin{aligned} B_{P}=\sum_{i,j,k\in\left\{ W,M \right\}} B_{ijk,P} \\ \left( EquationS43 \right) \end{aligned}$$

$$\begin{aligned} \frac{d}{dt}P_{C,\theta}=\left( b_{\theta}-1 \right)\cdot P_{C,\theta}\cdot\sum_{i,j,k\in\left\{ W,M \right\}} \left( k_{inf,\theta}\left( ijk \right)\cdot B_{ijk,P} \right) \\ -k_{12}\cdot P_{C,\theta}+k_{21}\cdot P_{P,\theta}-k_{el,\theta}\cdot P_{C,\theta} \\ \left( EquationS44 \right) \end{aligned}$$

$$\begin{aligned} \frac{d}{dt}P_{P,\theta}=k_{12}\cdot P_{C,\theta}-k_{21}\cdot P_{P,\theta} \\ \left( EquationS45 \right) \end{aligned}$$

$$\begin{aligned} \frac{d}{dt}B_{ijk,P}=k_{growth}\cdot B_{ijk,P}-P_{C,\theta}\cdot k_{inf,\theta}\left( ijk \right)\cdot B_{ijk,P}-k_{PD}\cdot B_{ijk,P}+k_{DP}\cdot B_{ijk,D} \\ -k_{death}\cdot B_{ijk,P}-\epsilon\cdot I\cdot B_{ijk,P}\cdot\frac{1}{1+\frac{B_{P}}{B_{50}}} \\ \left( EquationS46 \right) \end{aligned}$$

$$\begin{aligned} \frac{d}{dt}B_{ijk,D}=k_{PD}\cdot B_{ijk,P}-k_{DP}\cdot B_{ijk,D}-k_{death}\cdot B_{ijk,D} \\ \left( EquationS47 \right) \end{aligned}$$

$$\begin{aligned} \frac{d}{dt}I=\alpha\cdot B_{P}\cdot\frac{1}{1+\frac{B_{P}}{B_{50}'}}-\beta\cdot I \\ \left( EquationS48 \right) \end{aligned}$$

$$\begin{aligned} \phi_{WWW}=0 \\ \left( EquationS49 \right) \end{aligned}$$

$$\begin{aligned} B_{ijk,P}\left( 0 \right)=B_{0}\cdot\frac{exp\left( \phi_{ijk} \right)}{\sum_{x,y,z\in\left\{ W,M \right\}} exp\left( \phi_{xyz} \right)} \\ \left( EquationS50 \right) \end{aligned}$$

$$\begin{aligned} B_{ijk,D}\left( 0 \right)=0 \\ \left( EquationS51 \right) \end{aligned}$$

$$\begin{aligned} P_{C,\theta}\left( 0 \right)=Dose_{\theta} \\ \left( EquationS52 \right) \end{aligned}$$

$$\begin{aligned} P_{P,\theta}\left( 0 \right)=0 \\ \left( EquationS53 \right) \end{aligned}$$

$$\begin{aligned} I\left( 0 \right)=0 \\ \left( EquationS54 \right) \end{aligned}$$

($P_{C,\theta}$: Phage amount in the central compartment, $P_{P,\theta}$: Phage amount in the peripheral compartment; $I$: The immune sytem, $\alpha$: Induction rate constant for the immune system by total proliferative bacteria, $B_{50}'$: Load of proliferative bacteria needed to suppress the rate of immune system induction by half, $\beta$: Decay rate of the immune system, $\epsilon$: Killing rate of proliferative bacteria by the immune system, $B_{50}$: Load of proliferative bacteria needed to suppress the rate of immune system killing of bacteria by half, $Dose_{\theta}$: The administered phage dose with dose-to-lung scaling, $k_{12}$: Distribution rate constant from central to peripheral compartment, $k_{21}$: Distribution rate constant from peripheral to central compartment, $k_{el,\theta}$: phage elimination rate constant)

The calibration of the extra *in vivo* mouse PKPD parameters was conducted manually to give a good fit to the data. Some of the parameters estimated from *in vitro* studies were also recalibrated to better describe the *in vivo* PKPD measurements. The recalibrated parameters included the proliferative capacity of the target bacteria, the infection rate of PP-A, and the burst size of PP-A. Immune-related parameters were fixed based on literature values.(7) All other parameters were fixed to the values estimated from the *in vitro* data.

### *In vivo* mouse lung bacterial load experiment

Two sets of experiments were conducted: one aimed at assessing the impact of phage type or cocktail composition and the other focused on evaluating the dose response of the MP-A + PP-A phage cocktail on lung bacterial load in intranasally infected mice treated with phages. For the first experiment, groups were organized as follows: a control group (n=6), a $5\times{10}^{9}$ PFU/head MP-A treatment group (n=6), a $5\times{10}^{9}$ PFU/head PP-A treatment group (n=6), and a $5\times{10}^{9}$ PFU/head MP-A + PP-A treatment group (n=6). For the second experiment, groups were organized as follows: a control group (n=5), a ${10}^{7}$ PFU/head MP-A + PP-A treatment group (n=5), and a ${10}^{11}$ PFU/head MP-A + PP-A treatment group (n=5).

ICR mice were intranasally inoculated with 20 $\mu$L of prepared bacterial mixture. For the first experiment, bacterial doses of $6\times{10}^{7}$ CFU/head were administered, and for the second experiment, doses of $1.5\times{10}^{8}$ CFU/head were used. After a two-hour interval, either negative control saline or phage treatments were administered to the mice via the tail vein, with a volume of 200 $\mu$L. Upon the occurrence of the first mortality, all mice were euthanized, and lung tissues were collected. The time of first mortality was recorded as 21 hours for the first experiment and 20 hours for the second experiment. Bacterial quantification inside the lungs was performed using plating methods.

### Evaluating the efficacy of the cocktail

A total of $5\times{10}^{8}$ CFU/head of bacteria were inoculated intranasally into the mice to generate an acute pneumonia mouse model. For the control group, 0.2 mL of saline was injected into the tail vein and for the treatment group, $5\times{10}^{4}$, $5\times{10}^{6}$, $5\times{10}^{8}$, and $5\times{10}^{10}$ PFU/head of MP-A + PP-A cocktail was injected into the tail vein. Each control and treatment group had eight replicates. Survival was evaluated at 0, 24, 48, 72, and 96 hours.

### Quantification and statistical analysis

The specifics of quantification and statistical analysis are delineated in the figure legends. Analysis of variance (ANOVA) and Tukey’s honestly significant difference (HSD) test were conducted using the Python package SciPy v1.11.2.(8) The log-rank test for survival was performed and analysis and Kaplan-Meier estimators for survival probability were computed using the Python package Lifelines v0.27.7.(9) Noncompartmental analysis for phage pharmacokinetics was executed using Phoenix® WinNonlin® v8.3 (Certara, PA, USA). Nonlinear mixed-effects modeling of *in vitro* PKPD data was conducted using Monolix v2023R1 (Lixoft, Antony, France).

#

# SUPPLEMENTARY FIGURES


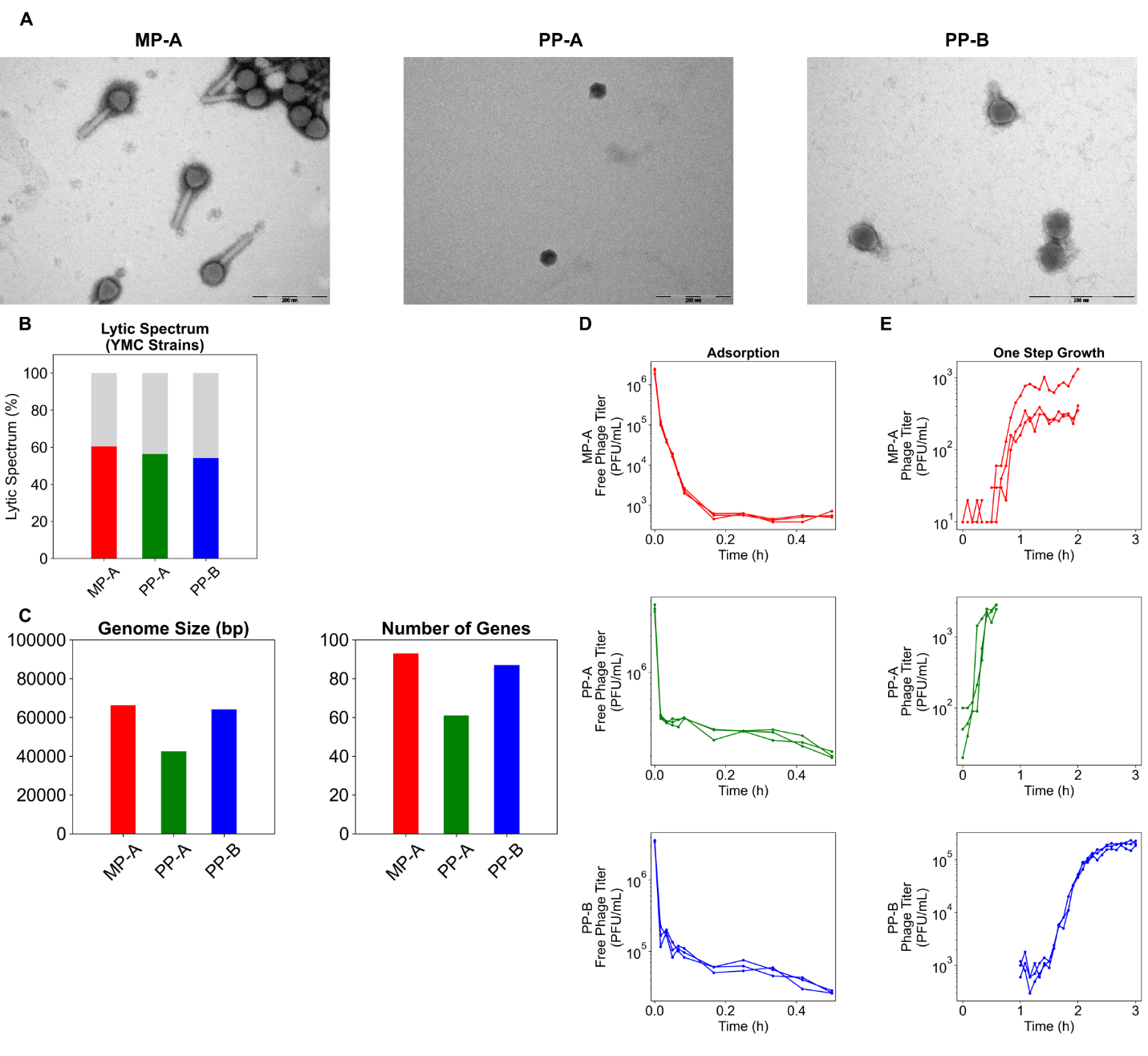


**FIG S1** Characterization of bacteriophages MP-A, PP-A, and PP-B. (A) TEM images revealed that all three phages belonged to the order *Caudovirales* (tailed bacteriophages). Specifically, MP-A is classified within the *Myoviridae* family, featuring long, contractile tails, whereas PP-A are PP-B are members of the *Podoviridae* family, characterized by short, non contractile tails. (B) The lytic activities of the phages were tested against the domestic YMC strains of *P. aeruginosa*; results revealed lytic spectra of 60%, 56%, and 54% for MP-A, PP-A, and PP-B, respectively. (C) Bioinformatics analysis determined genome sizes of 40,000-70,000 bp and gene counts of 50-100. (D) Adsorption tests using *P. aeruginosa* 15-4 revealed that all three phages achieved over 90% adsorption within 10 minutes, with a multiplicity of infection of 0.001 for MP-A and PP-A and 1.0 for PP-B. MP-A exhibited the highest adsorption efficiency, with less than 0.1% remaining unadsorbed after 30 minutes, followed by PP-B with less than 1% unadsorbed and PP-A with less than 10% unadsorbed. (E) One-step growth assays revealed the rapid lytic activity of PP-A initiating host cell lysis in under 10 minutes, whereas MP-A and PP-B had lysis times of approximately 40 minutes and 100 minutes, respectively. Burst size measurements showed that MP-A and PP-A had similar values of approximately 20 and 50, respectively, whereas PP-B exhibited a considerably larger burst size of approximately 200.


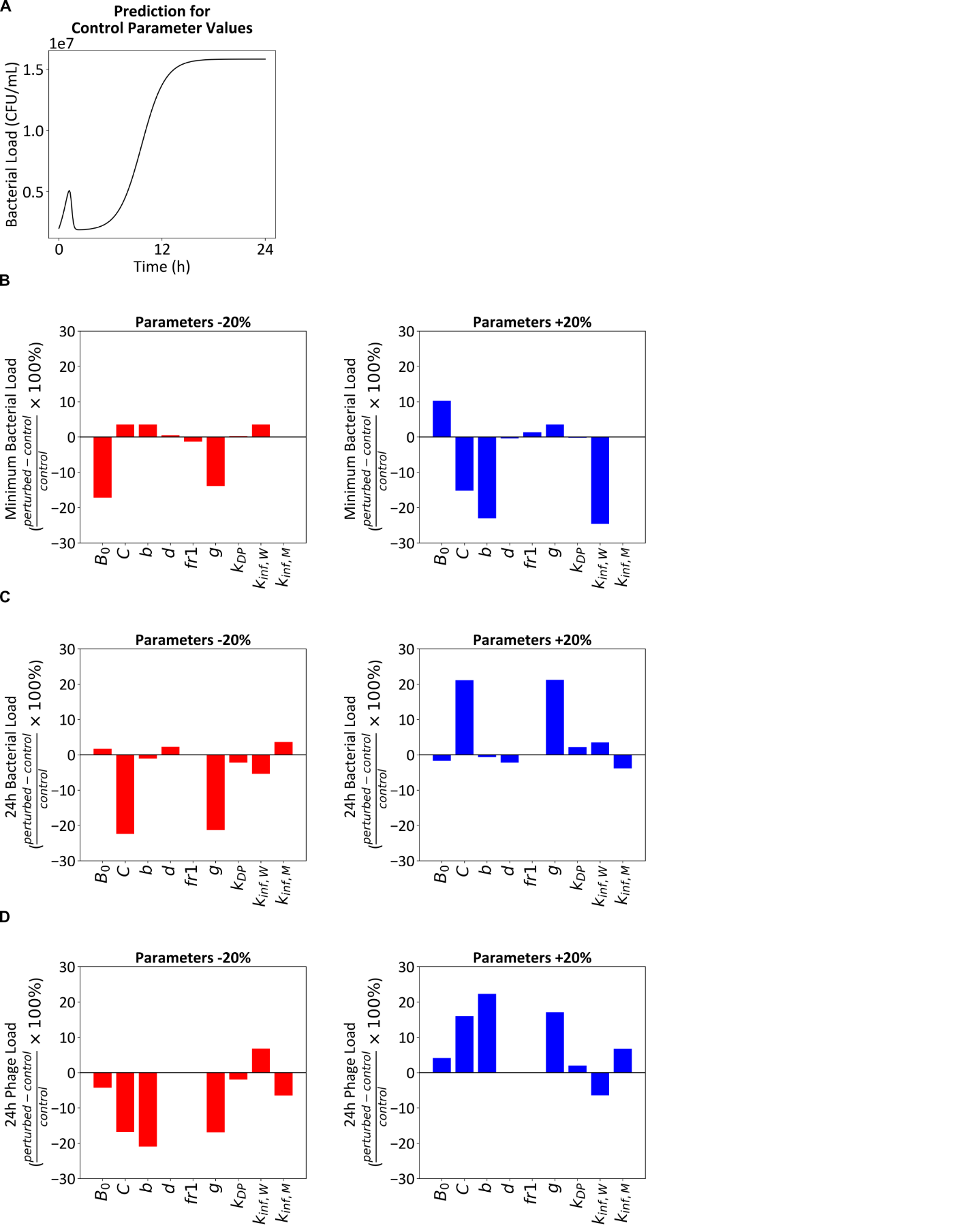


**FIG S2** Local sensitivity analysis for the single phage *in vitro* bacteria-phage dynamics model. (A) The control parameters yielding the predicted bacterial load time-course are $B_{0}$:0.01, $g$:1, $d$:0.01, $k_{FP}$:0.01, ${log}_{10}C$:7, $lgfr_{1}$:-5, ${log}_{10}k_{inf,W}$:-7, ${log}_{10}k_{inf,M}$:-9, and $b$:20. Local sensitivity analyses for (B) minimum bacterial load, (C) 24h bacterial load, and (D) 24h phage load were conducted. Relative changes (%) are plotted as bar graphs.


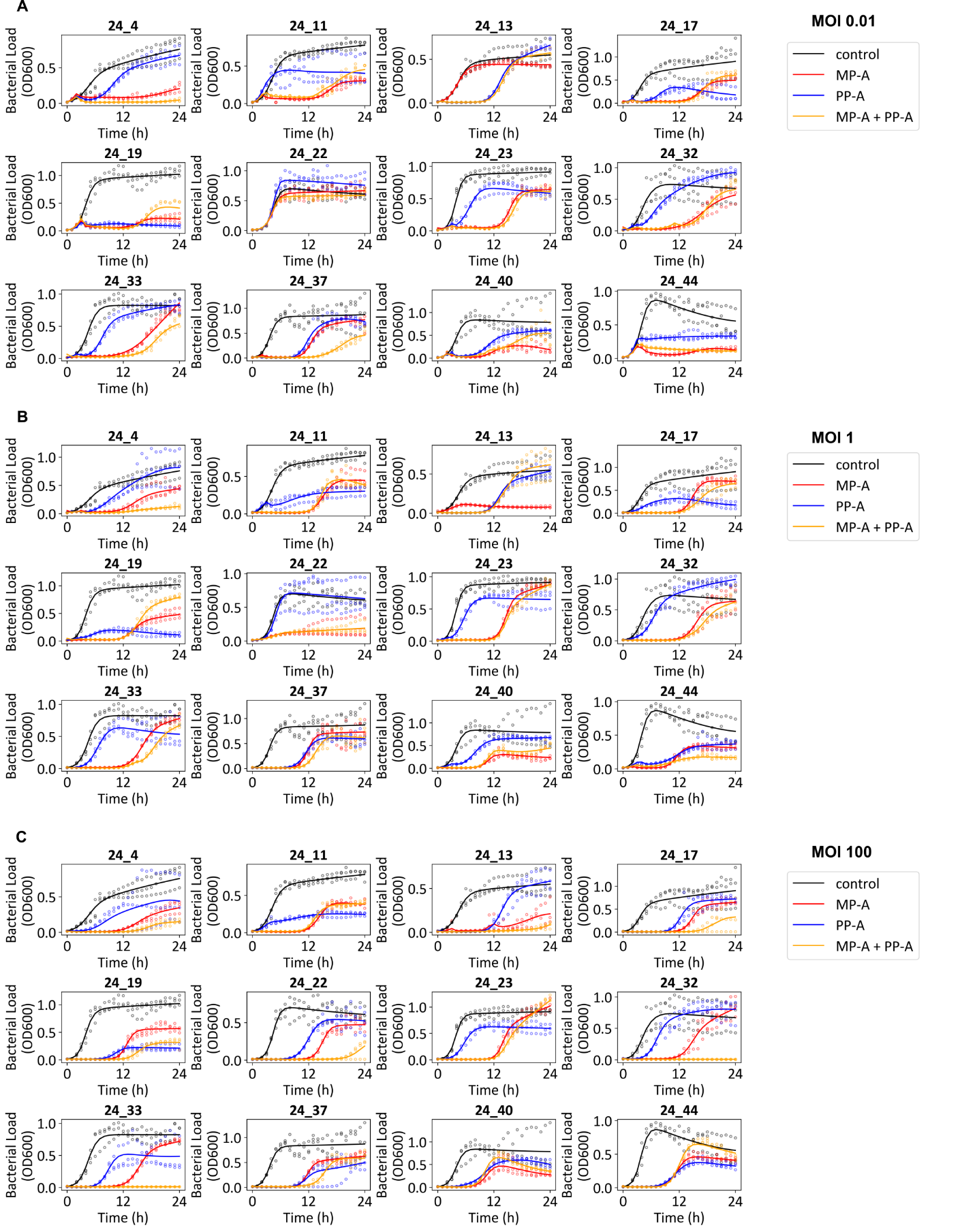


**FIG S3** Model fit of the extended bacteria-phage dynamics model across 12 additional bacterial strains. The *in vitro* kinetics assays were conducted under control, MP-A, PP-A, or combined MP-A + PP-A treatment conditions at multiplicities of infection (MOIs) of 0.01 (A), 1 (B), and 100 (C) for the 12 additional bacterial strains. Time-course experimental observations (circles) are compared with the corresponding mean individual predictions (solid lines), illustrating the goodness-of-fit of the model.


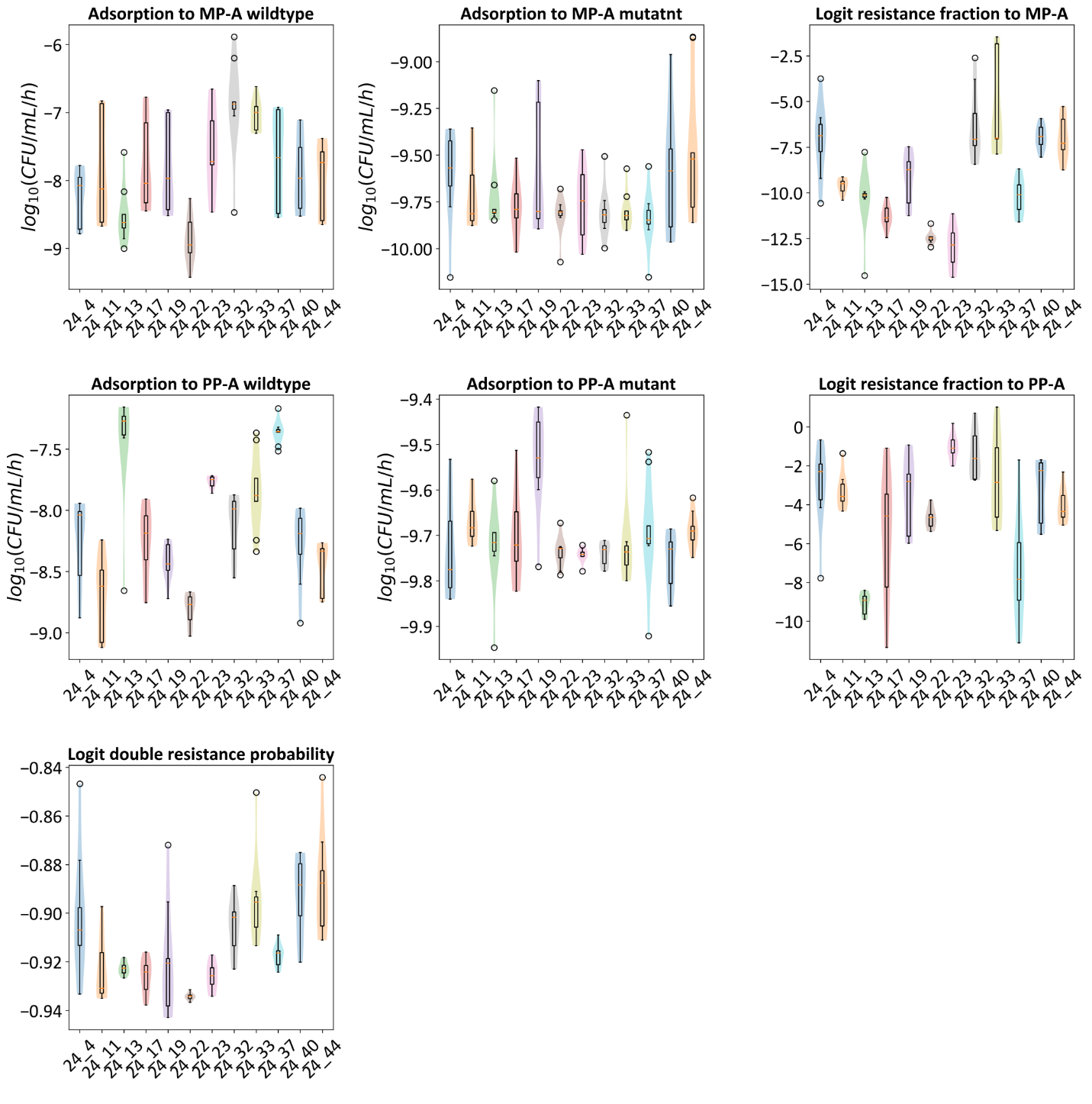


**FIG S4** Distributions of empirical Bayes estimates of individual model parameters across different bacterial strains. Violin plots overlaid with boxplots illustrate the distributions of empirical Bayes estimates for individual parameters across wells for each of the 12 bacterial strains.


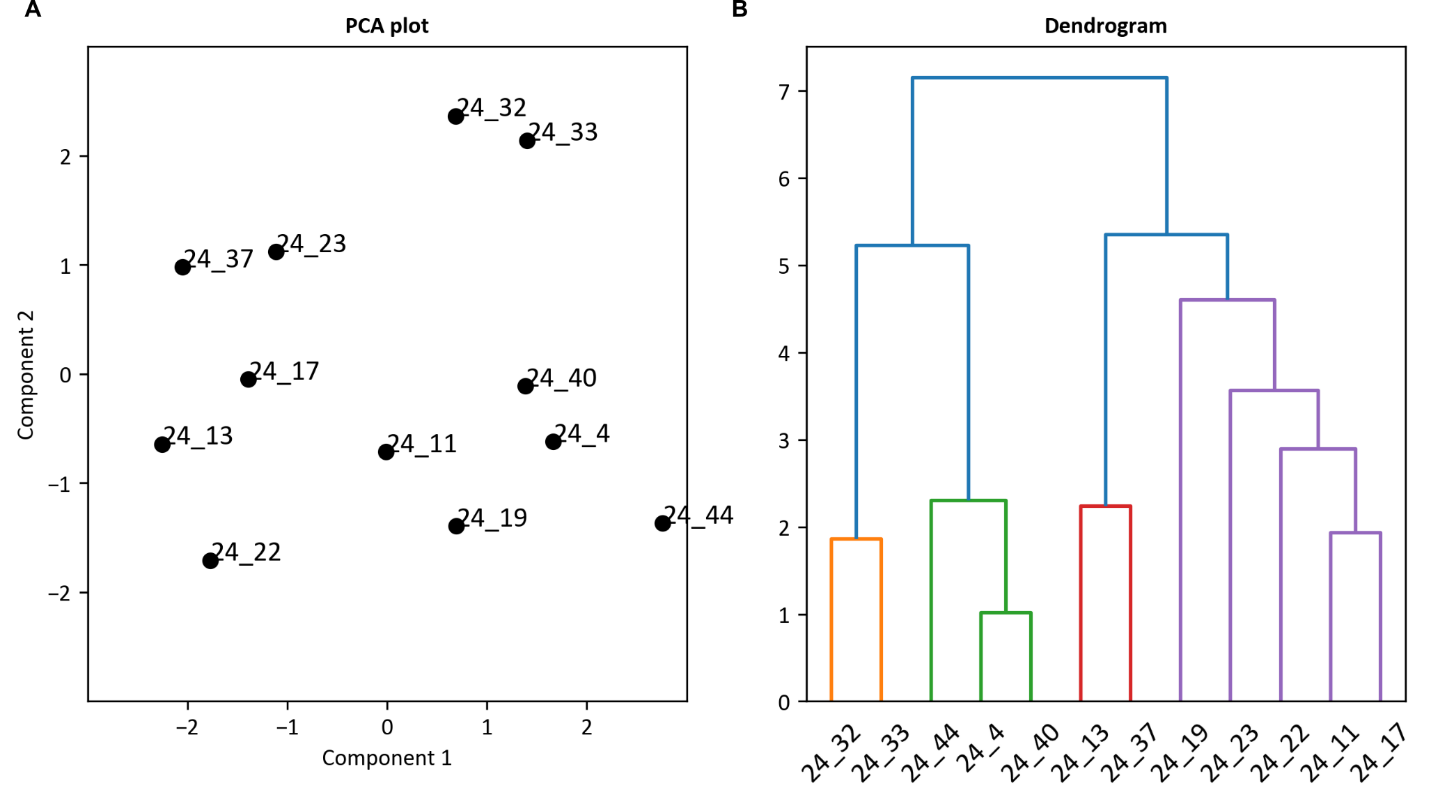


**FIG S5** Principal component analysis and clustering of bacterial strains based on model parameter values. (A) Bacterial strains were represented using the mean of their individual parameter estimates and projected onto two principal components via principal component analysis (PCA). (B) Strains were clustered based on their mean individual parameter estimates to identify patterns of similarity.


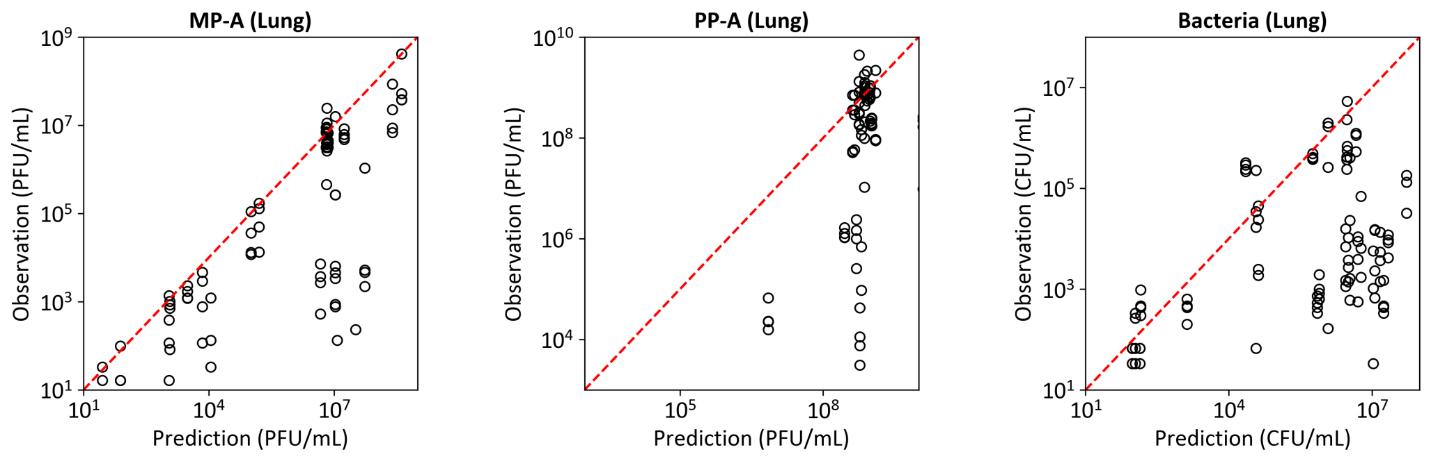


**FIG S6** Goodness-of-fit of extended *in vivo* bacteria-phage dynamics model without host immunity. All the parameter values were set equal to the extended *in vivo* bacteria-phage dynamics model with host immunity except for $\alpha$(induction rate constant for the immune system) and $\epsilon$(killing rate of proliferative bacteria by the immune system), which were set to zero to remove host immunity. The goodness-of-fit plot comparing observed and predicted values is presented. Only the datapoints observed before the first mortality for each dosing group is shown.

# SUPPLEMENTARY TABLES

**TABLE S1:** Parameter estimates of the in vitro mathematical model

| Parameter |  |  | Unit | Estimate ($RSE$) |
| --- | --- | --- | --- | --- |
| Inter Plate Variability |  |  |  |  |
| $\omega_{B_{0}}$ |  |  | CV(%) | 104.54 (3.73) |
| $\omega_{k_{growth}}$ |  |  | CV(%) | 32.06 (3.46) |
| $\omega_{k_{death}}$ |  |  | CV(%) | 207.38 (5.44) |
| $\omega_{k_{DP}}$ |  |  | CV(%) | 255.25 (4.92) |
| $\omega_{log_{10}\left( C \right)}$ |  |  | CV(%) | 3.25 (4.59) |
| $\omega_{\phi_{WWM}}$ |  |  | CV(%) | 48.33 (5.49) |
| $\omega_{\phi_{WMW}}$ |  |  | CV(%) | 24.53 (7.55) |
| $\omega_{\phi_{WMM}}$ |  |  | CV(%) | 32.15 (6.63) |
| $\omega_{\phi_{MWW}}$ |  |  | CV(%) | 16.05 (10.0) |
| $\omega_{\phi_{MWM}}$ |  |  | CV(%) | 45.29 (5.44) |
| $\omega_{\phi_{MMW}}$ |  |  | CV(%) | 32.67 (7.64) |
| $\omega_{\phi_{MMW}}$ |  |  | CV(%) | 36.33 (6.68) |
| $\omega_{log_{10}\left( k_{inf,W} \right)}$ |  | MP-A | CV(%) | 1.72 (13.8) |
|  |  | PP-A | CV(%) | 3.03 (10.4) |
|  |  | PP-B | CV(%) | 4.38 (9.68) |
| $\omega_{log_{10}\left( k_{inf,M} \right)}$ |  | MP-A | CV(%) | 0.96 (33.6) |
|  |  | PP-A | CV(%) | 3.12 (35.2) |
|  |  | PP-B | CV(%) | 4.53 (15.3) |
| $\omega_{b}$ |  | MP-A | CV(%) | 52.22 (7.56) |
|  |  | PP-A | CV(%) | 35.42 (10.7) |
|  |  | PP-B | CV(%) | 112.3 (8.22) |
| Correlations |  |  |  |  |
| $\rho_{k_{death},B_{0}}$ |  |  |  | -0.23 (28.4) |
| $\rho_{k_{growth},B_{0}}$ |  |  |  | -0.35 (12.9) |
| $\rho_{k_{DP},B_{0}}$ |  |  |  | -0.054 (118) |
| $\rho_{log_{10}\left( C \right),B_{0}}$ |  |  |  | 0.22 (26.3) |
| $\rho_{k_{growth},k_{death}}$ |  |  |  | 0.33 (17.8) |
| $\rho_{k_{DP},k_{death}}$ |  |  |  | -0.2 (40.0) |
| $\rho_{log_{10}\left( C \right),k_{death}}$ |  |  |  | 0.43 (14.7) |
| $\rho_{k_{DP},k_{growth}}$ |  |  |  | 0.1 (59.1) |
| $\rho_{log_{10}\left( C \right),k_{growth}}$ |  |  |  | -0.34 (15.2) |
| $\rho_{log_{10}\left( C \right),k_{DP}}$ |  |  |  | -0.67 (6.33) |
| Residual Variability |  |  |  |  |
| $err_{a}$ |  |  |  | 0.026 (0.753) |

^a^RSE(%): Relative Standard Error

**TABLE S2:** Parameter estimates for the 12 clinical strains

| Parameter |  |  | Unit | Estimate ($RSE$) |
| --- | --- | --- | --- | --- |
| Host Growth |  |  |  |  |
| $B_{0}$ | Initial nominal bacterial load |  | CFU/mL | 10^8^ (FIXED) |
| $k_{growth}$ | Bacterial growth rate constant |  | /h | 1.12 (3.75) |
| $k_{death}$ | Bacterial death rate constant |  | /h | 0.0099 (26.0) |
| $k_{DP}$ | Rate of conversion from dormant to proliferating subpopulations |  | /h | 0.0087 (33.1) |
| $log_{10}\left( C \right)$ | Proliferative capacity |  | $log_{10}CFU$ | 7.81 (0.246) |
| Phage-Bacteria Interaction |  |  |  |  |
| $log_{10}\left( k_{inf,MP-A,W} \right)$ | Log10-transformed adsorption rate constant of MP-A to wild type bacteria for strain 24_11 | Strain 24_11 | $log_{10}{{mL}/{CFU}}/h$ | -7.76 (2.93) |
| $\beta_{log_{10}\left( k_{inf,MP-A,W} \right),{24}_{13}}$ | Log ratio of Log10-transformed adsorption rate constant of MP-A to wild type bacteria compared to strain 24_11 | 24_13 |  | -0.74 (34.8) |
| $\beta_{log_{10}\left( k_{inf,MP-A,W} \right),{24}_{17}}$ |  | 24_17 |  | 0 (FIX) |
| $\beta_{log_{10}\left( k_{inf,MP-A,W} \right),{24}_{19}}$ |  | 24_19 |  | -1.34 (21.8) |
| $\beta_{log_{10}\left( k_{inf,MP-A,W} \right),{24}_{22}}$ |  | 24_22 |  | 0 (FIX) |
| $\beta_{log_{10}\left( k_{inf,MP-A,W} \right),{24}_{23}}$ |  | 24_23 |  | 0 (FIX) |
| $\beta_{log_{10}\left( k_{inf,MP-A,W} \right),{24}_{32}}$ |  | 24_32 |  | 0.95 (25.7) |
| $\beta_{log_{10}\left( k_{inf,MP-A,W} \right),{24}_{33}}$ |  | 24_33 |  | 0.66 (35.5) |
| $\beta_{log_{10}\left( k_{inf,MP-A,W} \right),{24}_{37}}$ |  | 24_37 |  | 0 (FIX) |
| $\beta_{log_{10}\left( k_{inf,MP-A,W} \right),{24}_{4}}$ |  | 24_4 |  | 0 (FIX) |
| $\beta_{log_{10}\left( k_{inf,MP-A,W} \right),{24}_{40}}$ |  | 24_40 |  | 0 (FIX) |
| $\beta_{log_{10}\left( k_{inf,MP-A,W} \right),{24}_{44}}$ |  | 24_44 |  | 0 (FIX) |
| $log_{10}\left( k_{inf,PP-A,W} \right)$ | Log10-transformed adsorption rate constant of PP-A to wild type bacteria for strain 24_11 | Strain 24_11 | $log_{10}{{mL}/{CFU}}/h$ | -8.74 (0.615) |
| $\beta_{log_{10}\left( k_{inf,PP-A,W} \right),{24}_{13}}$ | Log ratio of Log10-transformed adsorption rate constant of PP-A to wild type bacteria compared to strain 24_11 | 24_13 |  | 1.15 (10.9) |
| $\beta_{log_{10}\left( k_{inf,PP-A,W} \right),{24}_{17}}$ |  | 24_17 |  | 0.44 (26.0) |
| $\beta_{log_{10}\left( k_{inf,PP-A,W} \right),{24}_{19}}$ |  | 24_19 |  | 0 (FIX) |
| $\beta_{log_{10}\left( k_{inf,PP-A,W} \right),{24}_{22}}$ |  | 24_22 |  | 0 (FIX) |
| $\beta_{log_{10}\left( k_{inf,PP-A,W} \right),{24}_{23}}$ |  | 24_23 |  | 0.9 (15.2) |
| $\beta_{log_{10}\left( k_{inf,PP-A,W} \right),{24}_{32}}$ |  | 24_32 |  | 0.59 (20.3) |
| $\beta_{log_{10}\left( k_{inf,PP-A,W} \right),{24}_{33}}$ |  | 24_33 |  | 1.07 (13.2) |
| $\beta_{log_{10}\left( k_{inf,PP-A,W} \right),{24}_{37}}$ |  | 24_37 |  | 1.1 (12.1) |
| $\beta_{log_{10}\left( k_{inf,PP-A,W} \right),{24}_{4}}$ |  | 24_4 |  | 0.46 (25.0) |
| $\beta_{log_{10}\left( k_{inf,PP-A,W} \right),{24}_{40}}$ |  | 24_40 |  | 0.35 (32.5) |
| $\beta_{log_{10}\left( k_{inf,PP-A,W} \right),{24}_{44}}$ |  | 24_44 |  | 0 (FIX) |
| $log_{10}\left( k_{inf,MP-A,M} \right)$ | Log10-transformed adsorption rate constant of MP-A to mutant type bacteria |  | $log_{10}{{mL}/{CFU}}/h$ | -9.71 (0.843) |
| $log_{10}\left( k_{inf,PP-A,M} \right)$ | Log10-transformed adsorption rate constant of PP-A to mutant type bacteria |  | $log_{10}{{mL}/{CFU}}/h$ | -9.87 (0.330) |
| b | Burst size of phage | MP-A |  | 7.32 (13.2) |
|  |  | PP-A |  | 47.99 (6.82) |
| Subpopulation Structure |  |  |  |  |
| $\psi_{MP-A}$ | coefficient | Strain 24_11 |  | -9.86 (2.93) |
| $\beta_{MP-A,{24}_{13}}$ | Log ratio of the coefficient for the strain relative to the coefficient for strain24_11 | Strain 24_13 |  | 0 (FIX) |
| $\beta_{MP-A,{24}_{17}}$ |  | Strain 24_17 |  | 0 (FIX) |
| $\beta_{MP-A,{24}_{19}}$ |  | Strain 24_19 |  | -2.6 (48.6) |
| $\beta_{MP-A,{24}_{22}}$ |  | Strain 24_22 |  | 0 (FIX) |
| $\beta_{MP-A,{24}_{23}}$ |  | Strain 24_23 |  | -2.63 (27.0) |
| $\beta_{MP-A,{24}_{32}}$ |  | Strain 24_32 |  | 3.5 (18.4) |
| $\beta_{MP-A,{24}_{33}}$ |  | Strain 24_33 |  | 4.07 (16.1) |
| $\beta_{MP-A,{24}_{37}}$ |  | Strain 24_37 |  | 0 (FIX) |
| $\beta_{MP-A,{24}_{4}}$ |  | Strain 24_4 |  | 3.25 (21.0) |
| $\beta_{MP-A,{24}_{40}}$ |  | Strain 24_40 |  | 2.86 (23.3) |
| $\beta_{MP-A,{24}_{44}}$ |  | Strain 24_44 |  | 2.11 (30.5) |
| $\psi_{PP-A}$ | coefficient | Strain 24_11 |  | -3.41 (7.32) |
| $\beta_{PP-A,{24}_{13}}$ | Log ratio of the coefficient for the strain relative to the logit coefficient for strain24_11 | Strain 24_13 |  | -5.73 (13.0) |
| $\beta_{PP-A,{24}_{17}}$ |  | Strain 24_17 |  | -1.54 (45.2) |
| $\beta_{PP-A,{24}_{19}}$ |  | Strain 24_19 |  | 0 (FIX) |
| $\beta_{PP-A,{24}_{22}}$ |  | Strain 24_22 |  | 0 (FIX) |
| $\beta_{PP-A,{24}_{23}}$ |  | Strain 24_23 |  | 2.45 (28.1) |
| $\beta_{PP-A,{24}_{32}}$ |  | Strain 24_32 |  | 2.11 (32.9) |
| $\beta_{PP-A,{24}_{33}}$ |  | Strain 24_33 |  | 0 (FIX) |
| $\beta_{PP-A,{24}_{37}}$ |  | Strain 24_37 |  | -3.47 (20.4) |
| $\beta_{PP-A,{24}_{4}}$ |  | Strain 24_4 |  | 0 (FIX) |
| $\beta_{PP-A,{24}_{40}}$ |  | Strain 24_40 |  | 0 (FIX) |
| $\beta_{PP-A,{24}_{44}}$ |  | Strain 24_44 |  | 0 (FIX) |
| $\zeta$ | Double resistance fraction relative to the smaller fraction between MP-A resistance and PP-A resistance |  |  | 0.17 (17.8) |
| Baseline OD600 |  |  |  |  |
| $OD_{600,Baseline}$ | Baseline OD_600_ |  | OD_600_ | 0.0061 (6.37) |
| Inter-plate Variability |  |  |  |  |
| $\omega_{{OD}_{600,baseline}}$ |  |  | CV(%) | 22.74 (31.7) |
| $\omega_{k_{growth}}$ |  |  | CV(%) | 22.43 (12.1) |
| $\omega_{k_{death}}$ |  |  | CV(%) | 144.11 (19.8) |
| $\omega_{k_{DP}}$ |  |  | CV(%) | 340.97 (15.8) |
| $\omega_{{log}_{10}C}$ |  |  | CV(%) | 1.37 (13.7) |
| $\omega_{\psi_{MP-A}}$ |  |  | CV(%) | 16.79 (8.50) |
| $\omega_{\psi_{PP-A}}$ |  |  | CV(%) | 55.87 (7.29) |
| $\omega_{\zeta}$ |  |  | CV(%) | 95.05 (9.76) |
| $\omega_{{log}_{10}k_{inf,MP-A,W}}$ |  |  | CV(%) | 7.67 (7.56) |
| $\omega_{{log}_{10}k_{inf,PP-A,W}}$ |  |  | CV(%) | 3.29 (8.46) |
| $\omega_{{log}_{10}k_{inf,MP-A,M}}$ |  |  | CV(%) | 4.68 (15.4) |
| $\omega_{{log}_{10}k_{inf,PP-A,M}}$ |  |  | CV(%) | 1.77 (17.2) |
| $\omega_{b_{MP-A}}$ |  |  | CV(%) | 172.57 (8.40) |
| $\omega_{b_{PP-A}}$ |  |  | CV(%) | 49.96 (11.6) |
| $\omega_{B_{0}}$ |  |  | CV(%) | 287.63 (6.93) |
| Residual Variability |  |  |  |  |
| $err_{b}$ |  |  | CV(%) | 0.12 (2.65) |

^a^RSE(%): Relative Standard Error

**TABLE S3:** Cross-resistance probability for the 12 clinical strains

| Bacterial Strain | Fraction Resistant |  | Estimated Value | Expected Value Under Independent Mutations | Suggested Optimal Regimen Composition |
| --- | --- | --- | --- | --- | --- |
| 24_11 | MP-A |  | $5.22\times{10}^{-5}$ | - | MP-A |
|  | PP-A |  | $3.20\times{10}^{-2}$ | - |  |
|  | MP-A+PP-A |  | $8.67\times{10}^{-6}$  (high cross resistance) | $1.67\times{10}^{-6}$ |  |
| 24_13 | MP-A |  | $5.22\times{10}^{-5}$ | - | MP-A |
|  | PP-A |  | $1.07\times{10}^{-4}$ | - |  |
|  | MP-A+PP-A |  | $8.67\times{10}^{-6}$  (high cross resistance) | $5.60\times{10}^{-9}$ |  |
| 24_17 | MP-A |  | $5.22\times{10}^{-5}$ | - | MP-A |
|  | PP-A |  | $7.03\times{10}^{-3}$ | - |  |
|  | MP-A+PP-A |  | $8.67\times{10}^{-6}$  (high cross resistance) | $3.67\times{10}^{-7}$ |  |
| 24_19 | MP-A |  | $3.88\times{10}^{-6}$ | - | MP-A |
|  | PP-A |  | $3.20\times{10}^{-2}$ | - |  |
|  | MP-A+PP-A |  | $6.44\times{10}^{-7}$  (high cross resistance) | $1.24\times{10}^{-7}$ |  |
| 24_22 | MP-A |  | 5.22 $\times{10}^{-5}$ | - | MP-A |
|  | PP-A |  | $3.20\times{10}^{-2}$ | - |  |
|  | MP-A+PP-A |  | $8.67\times{10}^{-6}$  (high cross resistance) | $1.67\times{10}^{-6}$ |  |
| 24_23 | MP-A |  | $3.76\times{10}^{-6}$ | - | MP-A + PP-A |
|  | PP-A |  | $2.77\times{10}^{-1}$ | - |  |
|  | MP-A+PP-A |  | $6.25\times{10}^{-7}$  (low cross resistance) | $1.04\times{10}^{-6}$ |  |
| 24_32 | MP-A |  | $1.73\times{10}^{-3}$ |  | MP-A + PP-A |
|  | PP-A |  | $2.14\times{10}^{-1}$ |  |  |
|  | MP-A+PP-A |  | $2.87\times{10}^{-4}$  (low cross resistance) | $3.70\times{10}^{-4}$ |  |
| 24_33 | MP-A |  | $3.05\times{10}^{-3}$ | - | MP-A |
|  | PP-A |  | $3.20\times{10}^{-2}$ | - |  |
|  | MP-A+PP-A |  | $5.06\times{10}^{-4}$  (high cross resistance) | $9.75\times{10}^{-5}$ |  |
| 24_37 | MP-A |  | $5.22\times{10}^{-5}$ | - | MP-A |
|  | PP-A |  | $1.03\times{10}^{-3}$ | - |  |
|  | MP-A+PP-A |  | $8.67\times{10}^{-6}$  (high cross resistance) | $5.36\times{10}^{-8}$ |  |
| 24_4 | MP-A |  | $1.35\times{10}^{-3}$ | - | MP-A |
|  | PP-A |  | $3.20\times{10}^{-2}$ | - |  |
|  | MP-A+PP-A |  | $2.23\times{10}^{-4}$  (high cross resistance) | $4.30\times{10}^{-5}$ |  |
| 24_40 | MP-A |  | $9.11\times{10}^{-4}$ | - | MP-A |
|  | PP-A |  | $3.20\times{10}^{-2}$ | - |  |
|  | MP-A+PP-A |  | $1.51\times{10}^{-4}$  (high cross resistance) | $2.91\times{10}^{-5}$ |  |
| 24_44 | MP-A |  | $4.31\times{10}^{-4}$ | - | MP-A |
|  | PP-A |  | $3.20\times{10}^{-2}$ | - |  |
|  | MP-A+PP-A |  | $7.15\times{10}^{-5}$  (high cross resistance) | $1.38\times{10}^{-5}$ |  |

**Table S4:** Noncompartmental analysis of healthy mice PK

| MP-A, Serum |  |  |  |
| --- | --- | --- | --- |
|  | G2, ${10}^{7}$PFU/head | G3, ${10}^{9}$PFU/head | G4, ${10}^{11}$PFU/head |
| $C_{0}$ (PFU/mL) | $1.53\times{10}^{2}$ | $7.86\times{10}^{6}$ | $5.61\times{10}^{9}$ |
| $AUC_{last}$ (PFU$\cdot$h/mL) | $3.72\times{10}^{2}$ | $3.43\times{10}^{6}$ | $2.47\times{10}^{9}$ |
| Terminal Half-life (h) | 5.2 | 5.6 | 2.5 |
| ${C_{0}}/{Dose}$ (/mL) | $1.53\times{10}^{-5}$ | $7.86\times{10}^{-3}$ | $5.61\times{10}^{-2}$ |
| $AU{C_{last}}/{Dose}$ (h/mL) | $3.72\times{10}^{-5}$ | $3.43\times{10}^{-3}$ | $2.47\times{10}^{-2}$ |
| MP-A, Lung |  |  |  |
|  | G2, ${10}^{7}$PFU/head | G3, ${10}^{9}$PFU/head | G4, ${10}^{11}$PFU/head |
| $C_{0}$ (PFU/mL) | $2.50\times{10}^{1}$ | $2.03\times{10}^{5}$ | $9.63\times{10}^{8}$ |
| $AUC_{last}$ (PFU$\cdot$h/mL) | $1.21\times{10}^{3}$ | $1.69\times{10}^{6}$ | $3.11\times{10}^{9}$ |
| Terminal Half-life (h) | 25.6 | 17.5 | 10.4 |
| ${C_{0}}/{Dose}$ (/mL) | $2.50\times{10}^{-6}$ | $2.03\times{10}^{-4}$ | $9.63\times{10}^{-3}$ |
| $AU{C_{last}}/{Dose}$ (h/mL) | $1.21\times{10}^{-4}$ | $1.69\times{10}^{-3}$ | $3.11\times{10}^{-2}$ |
| Lung/Serum $C_{0}$ Ratio | 0.16 | 0.026 | 0.17 |
| PP-A, Serum |  |  |  |
|  | G2, ${10}^{7}$PFU/head | G3, ${10}^{9}$PFU/head | G4, ${10}^{11}$PFU/head |
| $C_{0}$ (PFU/mL) | $2.84\times{10}^{7}$ | $5.74\times{10}^{9}$ | $6.38\times{10}^{11}$ |
| $AUC_{last}$ (PFU$\cdot$h/mL) | $2.98\times{10}^{7}$ | $2.84\times{10}^{9}$ | $4.63\times{10}^{11}$ |
| Terminal Half-life (h) | 1.1 | 1.8 | 1.8 |
| ${C_{0}}/{Dose}$ (/mL) | $2.84\times{10}^{0}$ | $5.74\times{10}^{0}$ | $6.38\times{10}^{0}$ |
| $AU{C_{last}}/{Dose}$ (h/mL) | $2.98\times{10}^{0}$ | $2.84\times{10}^{0}$ | $4.63\times{10}^{0}$ |
| PP-A, Lung |  |  |  |
|  | G2, ${10}^{7}$PFU/head | G3, ${10}^{9}$PFU/head | G4, ${10}^{11}$PFU/head |
| $C_{0}$ (PFU/mL) | $4.21\times{10}^{5}$ | $4.78\times{10}^{7}$ | $5.13\times{10}^{9}$ |
| $AUC_{last}$ (PFU$\cdot$h/mL) | $5.78\times{10}^{5}$ | $4.82\times{10}^{7}$ | $1.50\times{10}^{10}$ |
| Terminal Half-life (h) | 8.7 | 18.1 | 6.9 |
| ${C_{0}}/{Dose}$ (/mL) | $4.21\times{10}^{-2}$ | $4.78\times{10}^{-2}$ | $5.13\times{10}^{-2}$ |
| $AU{C_{last}}/{Dose}$ (h/mL) | $5.78\times{10}^{-2}$ | $4.82\times{10}^{-2}$ | $1.50\times{10}^{-1}$ |
| Lung/Serum $C_{0}$ Ratio | 0.015 | 0.0083 | 0.0080 |

**TABLE S5**: Noncompartmental analysis of infected mice PK

| MP-A, Serum |  |  |  |
| --- | --- | --- | --- |
|  | G2, ${10}^{7}$PFU/head | G3, ${10}^{9}$PFU/head | G4, ${10}^{11}$PFU/head |
| $C_{0}$ (PFU/mL) | $1.41\times{10}^{3}$ | $1.87\times{10}^{9}$ | $2.18\times{10}^{11}$ |
| $AUC_{last}$ (PFU$\cdot$h/mL) | $2.62\times{10}^{3}$ | $4.81\times{10}^{8}$ | $6.06\times{10}^{10}$ |
| Terminal Half-life (h) | 3.7 | 5.8 | 6.5 |
| ${C_{0}}/{Dose}$ (/mL) | $1.41\times{10}^{-4}$ | $1.87\times{10}^{0}$ | $2.18\times{10}^{0}$ |
| $AU{C_{last}}/{Dose}$ (h/mL) | $2.62\times{10}^{-4}$ | $4.81\times{10}^{-1}$ | $6.06\times{10}^{-1}$ |
| Infected/Noninfected $AUC_{last}$ | 7.04 | 140.23 | 24.53 |
| MP-A, Lung |  |  |  |
|  | G2, ${10}^{7}$PFU/head | G3, ${10}^{9}$PFU/head | G4, ${10}^{11}$PFU/head |
| $C_{0}$ (PFU/mL) | $5.82\times{10}^{2}$ | $2.16\times{10}^{6}$ | $6.63\times{10}^{9}$ |
| $AUC_{last}$ (PFU$\cdot$h/mL) | $8.45\times{10}^{7}$ | $8.74\times{10}^{8}$ | $7.46\times{10}^{9}$ |
| Terminal Half-life (h) | NA | NA | 28.8 |
| ${C_{0}}/{Dose}$ (/mL) | $5.82\times{10}^{-5}$ | $2.16\times{10}^{-3}$ | $6.63\times{10}^{-2}$ |
| $AU{C_{last}}/{Dose}$ (h/mL) | $8.45\times{10}^{0}$ | $8.74\times{10}^{-1}$ | $7.46\times{10}^{-2}$ |
| Lung/Serum $C_{0}$ Ratio | 0.37 | 0.0012 | 0.03 |
| Infected/Noninfected $AUC_{last}$ | $6.98\times{10}^{5}$ | 517.16 | 2.40 |
| PP-A, Serum |  |  |  |
|  | G2, ${10}^{7}$PFU/head | G3, ${10}^{9}$PFU/head | G4, ${10}^{11}$PFU/head |
| $C_{0}$ (PFU/mL) | $4.79\times{10}^{7}$ | $3.34\times{10}^{9}$ | $2.40\times{10}^{11}$ |
| $AUC_{last}$ (PFU$\cdot$h/mL) | $3.59\times{10}^{9}$ | $6.40\times{10}^{9}$ | $2.66\times{10}^{12}$ |
| Terminal Half-life (h) | 1.9 | 2.8 | 3.5 |
| ${C_{0}}/{Dose}$ (/mL) | $4.79\times{10}^{0}$ | $3.34\times{10}^{0}$ | $2.40\times{10}^{0}$ |
| $AU{C_{last}}/{Dose}$ (h/mL) | $3.59\times{10}^{2}$ | $6.40\times{10}^{0}$ | $2.66\times{10}^{1}$ |
| Infected/Noninfected $AUC_{last}$ | 120.47 | 2.25 | 5.75 |
| PP-A, Lung |  |  |  |
|  | G2, ${10}^{7}$PFU/head | G3, ${10}^{9}$PFU/head | G4, ${10}^{11}$PFU/head |
| $C_{0}$ (PFU/mL) | $1.62\times{10}^{6}$ | $3.17\times{10}^{7}$ | $5.53\times{10}^{9}$ |
| $AUC_{last}$ (PFU$\cdot$h/mL) | $4.02\times{10}^{12}$ | $6.68\times{10}^{11}$ | $1.10\times{10}^{12}$ |
| Terminal Half-life (h) | NA | 16.8 | 44.6 |
| ${C_{0}}/{Dose}$ (/mL) | $1.62\times{10}^{-1}$ | $3.17\times{10}^{-2}$ | $5.53\times{10}^{-2}$ |
| $AU{C_{last}}/{Dose}$ (h/mL) | $4.02\times{10}^{5}$ | $6.68\times{10}^{2}$ | $1.10\times{10}^{1}$ |
| Lung/Serum $C_{0}$ Ratio | 0.034 | 0.0095 | 0.023 |
| Infected/Noninfected $AUC_{last}$ | $6.96\times{10}^{6}$ | $1.39\times{10}^{4}$ | 73.33 |

**Table S6:** Experimental setup for kinetic assay

| 96well Plate Number | Treatment Group |  |  | Number of Replicates |
| --- | --- | --- | --- | --- |
|  | Treatment Type | MOI | RATIO |  |
| 1 | Control | 0 | - | 3 |
|  | MP-A + PP-B | ${10}^{-5}$ | 3:1 | 3 |
|  |  |  | 1:1 | 3 |
|  |  |  | 1:3 | 3 |
|  |  | ${10}^{-3}$ | 3:1 | 3 |
|  |  |  | 1:1 | 3 |
|  |  |  | 1:3 | 3 |
|  |  | ${10}^{-1}$ | 3:1 | 3 |
|  |  |  | 1:1 | 3 |
|  |  |  | 1:3 | 3 |
| 2 | Control | 0 | - | 3 |
|  | MP-A + PP-B | ${10}^{-7}$ | 3:1 | 3 |
|  |  |  | 1:1 | 3 |
|  |  |  | 1:3 | 3 |
|  |  | ${10}^{-5}$ | 3:1 | 3 |
|  |  |  | 1:1 | 3 |
|  |  |  | 1:3 | 3 |
|  |  | ${10}^{-3}$ | 3:1 | 3 |
|  |  |  | 1:1 | 3 |
|  |  |  | 1:3 | 3 |
|  |  | ${10}^{-1}$ | 3:1 | 3 |
|  |  |  | 1:1 | 3 |
|  |  |  | 1:3 | 3 |
| 3 | Control | 0 | - | 3 |
|  | PP-A + PP-B | ${10}^{-7}$ | 3:1 | 3 |
|  |  |  | 1:1 | 3 |
|  |  |  | 1:3 | 3 |
|  |  | ${10}^{-5}$ | 3:1 | 3 |
|  |  |  | 1:1 | 3 |
|  |  |  | 1:3 | 3 |
|  |  | ${10}^{-3}$ | 3:1 | 3 |
|  |  |  | 1:1 | 3 |
|  |  |  | 1:3 | 3 |
|  |  | ${10}^{-1}$ | 3:1 | 3 |
|  |  |  | 1:1 | 3 |
|  |  |  | 1:3 | 3 |
| 4 | Control | 0 | - | 3 |
|  | MP-A + PP-A | ${10}^{-7}$ | 3:1 | 3 |
|  |  |  | 1:1 | 3 |
|  |  |  | 1:3 | 3 |
|  |  | ${10}^{-5}$ | 3:1 | 3 |
|  |  |  | 1:1 | 3 |
|  |  |  | 1:3 | 3 |
|  |  | ${10}^{-3}$ | 3:1 | 3 |
|  |  |  | 1:1 | 3 |
|  |  |  | 1:3 | 3 |
|  |  | ${10}^{-1}$ | 3:1 | 3 |
|  |  |  | 1:1 | 3 |
|  |  |  | 1:3 | 3 |
| 5 | Control | 0 | - | 3 |
|  | PP-B | ${10}^{-7}$ | - | 3 |
|  |  | ${10}^{-5}$ | - | 3 |
|  |  | ${10}^{-3}$ | - | 3 |
|  |  | ${10}^{-1}$ | - | 3 |
|  | MP-A+PP-B | ${10}^{-7}$ | 1:1 | 3 |
|  |  | ${10}^{-5}$ | 1:1 | 3 |
|  |  | ${10}^{-3}$ | 1:1 | 3 |
|  |  | ${10}^{-1}$ | 1:1 | 3 |
| 6 | Control | 0 | - | 3 |
|  | MP-A | ${10}^{-7}$ | - | 3 |
|  |  | ${10}^{-5}$ | - | 3 |
|  |  | ${10}^{-3}$ | - | 3 |
|  |  | ${10}^{-1}$ | - | 3 |
|  | PP-A + PP-B | ${10}^{-7}$ | 1:1 | 3 |
|  |  | ${10}^{-5}$ | 1:1 | 3 |
|  |  | ${10}^{-3}$ | 1:1 | 3 |
|  |  | ${10}^{-1}$ | 1:1 | 3 |
| 7 | Control | 0 | - | 3 |
|  | MP-A | ${10}^{-7}$ | - | 3 |
|  |  | ${10}^{-5}$ | - | 3 |
|  |  | ${10}^{-3}$ | - | 3 |
|  |  | ${10}^{-1}$ | - | 3 |
|  | MP-A + PP-A | ${10}^{-7}$ | 1:1 | 3 |
|  |  | ${10}^{-5}$ | 1:1 | 3 |
|  |  | ${10}^{-3}$ | 1:1 | 3 |
|  |  | ${10}^{-1}$ | 1:1 | 3 |
| 8 | Control | 0 | - | 3 |
|  | PP-A | ${10}^{-7}$ | - | 3 |
|  |  | ${10}^{-5}$ | - | 3 |
|  |  | ${10}^{-3}$ | - | 3 |
|  |  | ${10}^{-1}$ | - | 3 |
|  | MP-A + PP-A + PP-B | ${10}^{-7}$ | 1:1:1 | 3 |
|  |  | ${10}^{-5}$ | 1:1:1 | 3 |
|  |  | ${10}^{-3}$ | 1:1:1 | 3 |
|  |  | ${10}^{-1}$ | 1:1:1 | 3 |
| 9 | Control | 0 | - | 6 |
|  | PP-B | ${10}^{-7}$ | - | 3 |
|  |  | ${10}^{-5}$ | - | 3 |
|  |  | ${10}^{-3}$ | - | 3 |
|  |  | ${10}^{-1}$ | - | 3 |
|  |  | ${10}^{0}$ | - | 3 |
|  |  | ${10}^{1}$ | - | 3 |
|  | MP-A | ${10}^{-7}$ | - | 3 |
|  |  | ${10}^{-5}$ | - | 3 |
|  |  | ${10}^{-3}$ | - | 3 |
|  |  | ${10}^{-1}$ | - | 3 |
|  |  | ${10}^{0}$ | - | 3 |
|  |  | ${10}^{1}$ | - | 3 |
|  | PP-A | ${10}^{-7}$ | - | 3 |
|  |  | ${10}^{-5}$ | - | 3 |
|  |  | ${10}^{-3}$ | - | 3 |
|  |  | ${10}^{-1}$ | - | 3 |
|  |  | ${10}^{0}$ | - | 3 |
|  |  | ${10}^{1}$ | - | 3 |
| 10 | Control | 0 | - | 6 |
|  | PP-B | ${10}^{-7}$ | - | 3 |
|  |  | ${10}^{-5}$ | - | 3 |
|  |  | ${10}^{-3}$ | - | 3 |
|  |  | ${10}^{-1}$ | - | 3 |
|  |  | ${10}^{0}$ | - | 3 |
|  |  | ${10}^{1}$ | - | 3 |
|  | MP-A | ${10}^{-7}$ | - | 3 |
|  |  | ${10}^{-5}$ | - | 3 |
|  |  | ${10}^{-3}$ | - | 3 |
|  |  | ${10}^{-1}$ | - | 3 |
|  |  | ${10}^{0}$ | - | 3 |
|  |  | ${10}^{1}$ | - | 3 |
|  | PP-A | ${10}^{-7}$ | - | 3 |
|  |  | ${10}^{-5}$ | - | 3 |
|  |  | ${10}^{-3}$ | - | 3 |
|  |  | ${10}^{-1}$ | - | 3 |
|  |  | ${10}^{0}$ | - | 3 |
|  |  | ${10}^{1}$ | - | 3 |
| 11 | Control | 0 | - | 3 |
|  | MP-A + PP-A | ${10}^{-6}$ | 1:1 | 3 |
|  |  | ${10}^{-4}$ | 1:1 | 3 |
|  |  | ${10}^{-2}$ | 1:1 | 3 |
|  |  | ${10}^{0}$ | 1:1 | 3 |
|  |  | ${10}^{1}$ | 1:1 | 3 |
|  |  | ${10}^{2}$ | 1:1 | 3 |
| 12 | Control | 0 | - | 3 |
|  | PP-B | ${10}^{-6}$ | - | 3 |
|  |  | ${10}^{-4}$ | - | 3 |
|  |  | ${10}^{-2}$ | - | 3 |
|  |  | ${10}^{0}$ | - | 3 |
|  |  | ${10}^{1}$ | - | 3 |
|  |  | ${10}^{2}$ | - | 3 |
|  | MP-A | ${10}^{-6}$ | - | 3 |
|  |  | ${10}^{-4}$ | - | 3 |
|  |  | ${10}^{-2}$ | - | 3 |
|  |  | ${10}^{0}$ | - | 3 |
|  |  | ${10}^{1}$ | - | 3 |
|  |  | ${10}^{2}$ | - | 3 |
|  | PP-A | ${10}^{-6}$ | - | 3 |
|  |  | ${10}^{-4}$ | - | 3 |
|  |  | ${10}^{-2}$ | - | 3 |
|  |  | ${10}^{0}$ | - | 3 |
|  |  | ${10}^{1}$ | - | 3 |
|  |  | ${10}^{2}$ | - | 3 |
| 13 | Control | 0 | - | 3 |
|  | MP-A + PP-A + PP-B | ${10}^{-6}$ | 1:1:1 | 3 |
|  |  | ${10}^{-4}$ | 1:1:1 | 3 |
|  |  | ${10}^{-2}$ | 1:1:1 | 3 |
|  |  | ${10}^{0}$ | 1:1:1 | 3 |
|  |  | ${10}^{1}$ | 1:1:1 | 3 |
|  |  | ${10}^{2}$ | 1:1:1 | 3 |
| 14 | Control | 0 | - | 3 |
|  | MP-A + PP-B | ${10}^{-6}$ | 1:1 | 3 |
|  |  | ${10}^{-4}$ | 1:1 | 3 |
|  |  | ${10}^{-2}$ | 1:1 | 3 |
|  |  | ${10}^{0}$ | 1:1 | 3 |
|  |  | ${10}^{1}$ | 1:1 | 3 |
|  |  | ${10}^{2}$ | 1:1 | 3 |
|  | PP-A + PP-B | ${10}^{-6}$ | 1:1 | 3 |
|  |  | ${10}^{-4}$ | 1:1 | 3 |
|  |  | ${10}^{-2}$ | 1:1 | 3 |
|  |  | ${10}^{0}$ | 1:1 | 3 |
|  |  | ${10}^{1}$ | 1:1 | 3 |
|  |  | ${10}^{2}$ | 1:1 | 3 |

# SUPPLEMENTARY REFERENCES

1. Bolger AM, Lohse M, Usadel B. 2014. Trimmomatic: a flexible trimmer for Illumina sequence data. Bioinformatics 30:2114–2120.

2. Prjibelski A, Antipov D, Meleshko D, Lapidus A, Korobeynikov A. 2020. Using SPAdes De Novo Assembler. Current Protocols in Bioinformatics 70:e102.

3. Seemann T. 2014. Prokka: rapid prokaryotic genome annotation. Bioinformatics 30:2068–2069.

4. Gurevich A, Saveliev V, Vyahhi N, Tesler G. 2013. QUAST: quality assessment tool for genome assemblies. Bioinformatics 29:1072–1075.

5. Chan PP, Lin BY, Mak AJ, Lowe TM. 2021. tRNAscan-SE 2.0: improved detection and functional classification of transfer RNA genes. Nucleic Acids Research 49:9077–9096.

6. Schneider CA, Rasband WS, Eliceiri KW. 2012. NIH Image to ImageJ: 25 years of image analysis. Nat Methods 9:671–675.

7. Roach DR, Leung CY, Henry M, Morello E, Singh D, Di Santo JP, Weitz JS, Debarbieux L. 2017. Synergy between the Host Immune System and Bacteriophage Is Essential for Successful Phage Therapy against an Acute Respiratory Pathogen. Cell Host & Microbe 22:38-47.e4.

8. Virtanen P, Gommers R, Oliphant TE, Haberland M, Reddy T, Cournapeau D, Burovski E, Peterson P, Weckesser W, Bright J, van der Walt SJ, Brett M, Wilson J, Millman KJ, Mayorov N, Nelson ARJ, Jones E, Kern R, Larson E, Carey CJ, Polat İ, Feng Y, Moore EW, VanderPlas J, Laxalde D, Perktold J, Cimrman R, Henriksen I, Quintero EA, Harris CR, Archibald AM, Ribeiro AH, Pedregosa F, van Mulbregt P. 2020. SciPy 1.0: fundamental algorithms for scientific computing in Python. Nat Methods 17:261–272.

9. Davidson-Pilon C. 2019. lifelines: survival analysis in Python. Journal of Open Source Software 4:1317.
